# Central sensorimotor integration assessment reveals deficits in standing balance control in people with chronic mild traumatic brain injury

**DOI:** 10.3389/fneur.2022.897454

**Published:** 2022-10-21

**Authors:** Kody R. Campbell, Laurie A. King, Lucy Parrington, Peter C. Fino, Prokopios Antonellis, Robert J. Peterka

**Affiliations:** ^1^Department of Neurology, Oregon Health and Science University, Portland, OR, United States; ^2^National Center for Rehabilitative Auditory Research (NCRAR), VA Portland Health Care System, Portland, OR, United States; ^3^Department of Dietetics, Human Nutrition and Sport, La Trobe University, Melbourne, VIC, Australia; ^4^Department of Health and Kinesiology, University of Utah, Salt Lake City, UT, United States

**Keywords:** mTBI, concussion, sensory integration, balance, vestibular, vision, time delay, SOT (Sensory Organization Test)

## Abstract

Imbalance is common following mild Traumatic Brain Injury (mTBI) and can persist months after the initial injury. To determine if mTBI subjects with chronic imbalance differed from healthy age- and sex-matched controls (HCs) we used both the Central SensoriMotor Integration (CSMI) test, which evaluates sensory integration, time delay, and motor activation properties and the standard Sensory Organization Test (SOT). Four CSMI conditions evoked center-of-mass sway in response to: surface tilts with eyes closed (SS/EC), surface tilts with eyes open viewing a fixed visual surround (SS/EO), visual surround tilts with eyes open standing on a fixed surface (VS/EO), and combined surface and visual tilts with eyes open (SS+VS/EO). The mTBI participants relied significantly more on visual cues during the VS/EO condition compared to HCs but had similar reliance on combinations of vestibular, visual, and proprioceptive cues for balance during SS/EC, SS/EO, and SS+VS/EO conditions. The mTBI participants had significantly longer time delays across all conditions and significantly decreased motor activation relative to HCs across conditions that included surface-tilt stimuli with a sizeable subgroup having a prominent increase in time delay coupled with reduced motor activation while demonstrating no vestibular sensory weighting deficits. Decreased motor activation compensates for increased time delay to maintain stability of the balance system but has the adverse consequence that sensitivity to both internal (e.g., sensory noise) and external disturbances is increased. Consistent with this increased sensitivity, SOT results for mTBI subjects showed increased sway across all SOT conditions relative to HCs with about 45% of mTBI subjects classified as having an “Aphysiologic” pattern based on published criteria. Thus, CSMI results provided a plausible physiological explanation for the aphysiologic SOT pattern. Overall results suggest that rehabilitation that focuses solely on sensory systems may be incomplete and may benefit from therapy aimed at enhancing rapid and vigorous responses to balance perturbations.

## Introduction

Persistent symptoms in people recovering from a mild traumatic brain injury (mTBI) months after the initial head injury can directly impact daily function and quality of life (1–3). A common, persistent symptom is imbalance, with approximately 28% of people still reporting problems more than a year following their injury (4). To maintain balance, sensory information from visual, vestibular, and proprioceptive/somatosensory systems must be integrated in the brain (5, 6). Recent evidence suggests that those with chronic mTBI (mTBI symptoms > 3 months after injury) have largely normal peripheral vestibular and visual system function based on clinical tests of vestibular reflexes and ocular motor behavior (7, 8). However, standard clinical tests of balance function, such as the Balance Error Scoring System (BESS) test and the Sensory Organization Test (SOT) have primarily demonstrated impaired balance function acutely after mTBI but have been mainly applied in early periods (<3 months) following brain injury (9–12).

Impaired balance performance is indicated by generalized increases in body sway, assessed using either successful completion of trials (no falls) or center-of-pressure (CoP) measures of sway variability on trials that alter the availability of accurate sensory orientation cues (9–12). That is, assessments are typically based on the evaluation of “condition-dependent sway.” The SOT battery uses condition-dependent sway to assess the integrity of the sensory systems contributing to balance control by measuring CoP displacements while standing in conditions that alter the availability and/or accuracy of sensory cues (eyes open, eyes closed, sway-referencing of the support surface and/or visual surround) with an overall “composite score” summarizing results from all the test conditions. Studies using the SOT have shown that people acutely (<2 weeks post-injury) and subacutely (2–12 weeks post-injury) recovering from mTBI had overall deficits in sensorimotor integration, as indicated by lower composite scores, and deficits in utilizing vestibular and visual information for balance (9, 13, 14).

Less common are assessments of mTBI-related balance deficits using “stimulus-evoked sway” measures derived from sway responses to stimuli applied to various sensory modalities to identify their influence on balance. For example, larger magnitude CoP sway was evoked in mTBI subjects than healthy controls (HC) when exposed to a rotating visual scene (15–17). In these studies, increased responsiveness to visual motion was interpreted as indicating abnormal visual-vestibular processing since mTBI subjects apparently had reduced ability to use vestibular orientation cues to suppress the effects of the visual stimulus. Similar results were reported in an earlier study (18). Another recent study used combinations of sinusoidal dynamic visual motion, galvanic vestibular stimulation, and proprioceptive Achilles heel vibration to perturb balance (19). In this study, sensitivity measures (also referred to as “gain” measures) were based on the ratio of center-of-mass (CoM) sway amplitude to the amplitude of the applied stimuli with these ratios assumed to be proportional to “sensory weights” which represent the relative contributions of the sensory systems to balance control. Results showed that visual and vestibular gains were highest in subjects with a recent mTBI, were lower in those with mTBI histories, and were lowest in HC subjects. Additionally, their analysis also measured “residual power” which characterized the magnitude of body sway that was not correlated with the applied stimulus. Residual power provided another indicator of sway variability that distinguished subjects with recent mTBI, who had high values of residual power, from the other two groups.

While the abovementioned methods for measuring balance performance have proved useful in assessing deficits caused by mTBI and tracking the time course of recovery, these methods focused on the sensory contribution to balance and did not assess other key elements of the balance control system that may also be affected by head injury. Specifically, the balance control system is understood to be organized as a closed-loop feedback control system comprised of peripheral sensory components, a central sensory integration mechanism, a motor activation mechanism that generates stabilizing joint torques as a function of the sensory-derived estimate of body orientation, and time delays from central processing, neural transmission, and muscle activation (6, 20, 21). Dysfunction in any part of the closed-loop feedback control system may contribute to balance deficits after mTBI. Previous studies of balance deficits in mTBI have not used methods that can quantify the various components of the balance control system. We developed the Central Sensorimotor Integration (CSMI) test that uses stimulus-evoked sway to provide measurements of sensory, time delay, and motor aspects of the balance control system (6, 20, 22). CSMI test methods have been used to characterize balance control in several populations including unilateral and bilateral vestibular loss patients, patients with other less defined vestibular disorders, people with Parkinson's Disease, healthy elderly and elderly with impaired balance, and patient populations with cataract and polyneuropathy (6, 23–27).

We hypothesized that people with chronic imbalance complaints after mTBI would differ significantly from HCs on visual-vestibular processing for balance control quantified from CSMI testing. However, proper analysis of the balance system requires the use of methods, such as the CSMI analysis, that are appropriate for the identification of a closed-loop system to correctly attribute the measured stimulus-response behavior, such as increased responsiveness to an applied stimulus, to a specific mechanism within the balance system (28, 29). Of relevance to our results, increased responsiveness to an applied balance perturbation can arise from decreased motor activation rather than altered reliance on sensory cues. Because the CSMI parameters represent functionally distinct components of the balance control system, identification of those components that differ from healthy controls gives insight into mTBI-associated imbalance. A clearer understanding of the underlying mechanism of balance dysfunction offers the potential for developing targeted rehabilitation programs to improve balance control.

## Materials and methods

### Participants

People with chronic mTBI symptoms (*n* = 52) and healthy age- and sex-matched controls (HC; *n* = 58) participated in the study (Table 1). All study participants gave written informed consent and the Oregon Health & Science University and Veterans Affairs Portland Health Care System joint institutional review board approved recruitment procedures and experimental protocols. Participants were recruited as part of a larger study on assessing and providing rehabilitation of balance after mTBI (ClinicalTrials.gov identifier: NCT02748109). Broader study inclusion/exclusion criteria, protocol, definitions for mTBI, and mTBI diagnosis confirmation were detailed previously (30). Briefly, participants were included if they: (1) were between 18 and 60 years old, (2) had minimal-to-no cognitive deficits indicated by a Short Blessed Test score ≤ 8, and (3) were >3 months post mTBI with unresolved balance complaints (for mTBI group); or (4) had no history of brain injury in the prior year (for HC group). Participants were excluded if they had: (1) a previous or current musculoskeletal injury, surgery, medication, or neurological illness that would influence balance, (2) a self-reported pre-existing peripheral vestibular/oculomotor pathology for the mTBI participants (3) a self-reported pre-existing or current peripheral vestibular/oculomotor pathology for control participants, (4) moderate to severe substance abuse. Neuroimaging was not performed as part of the study design. We used the Veterans Health Affairs and Department of Defense criteria for mTBI diagnosis (31). All diagnoses of mTBI were confirmed by medical record review and structured interview during participant screening and recruitment. When a medical record was unavailable an Oregon Health & Science University physician had a clinical visit with the prospective participant to confirm mTBI diagnosis.

**Table 1:** Participant demographics for the healthy control (HC) group and the chronic mild traumatic brain injury (mTBI) group.

	**HC (*n* = 58)**	**mTBI (*n*= 52)**	**Group difference *p*-value**
**Age (years)**	36.89 (12.68)	38.62 (10.92)	
**Height (cm)**	171.01 (9.57)	172.01 (9.44)	0.5849^a^
**Mass (kg)**	74.86 (19.26)	81.86 (19.72)	0.0625^a^
**Sex (M/F)**	23 M / 35 F	17 M / 35 F	
**DHI total score (out of 100)**	**0.56 (2.12)**	**36.54 (19.62)**	**<0.0001** ^**b**^
**NSI total score (out of 88)**	**3.98 (4.11)**	**35.17 (14.94)**	**<0.0001** ^**b**^
**Beck's depression inventory (out of 28)**	**2.60 (3.16)**	**17.44 (9.58)**	**<0.0001** ^**b**^
**Post–traumatic stress disorder checklist (17 to 85)**	**20.60 (6.11)**	**41.19 (15.51)**	**<0.0001** ^**b**^
**ANAM composite score (−4 to** **+4)**	**0.22 (0.84)**	**−0.65 (1.24)**	**<0.0001** ^**a**^
**SOT composite score (out of 100)**	**74.5 (8.2)**	**57.7 (19.5)**	**<0.0001** ^**a**^
**Days since injury (median and 1st Quartile, 3rd Quartile)**		398 (216, 932)	
**Injury mechanism (N and Percent)**			
**Blast**		1 (1.9%)	
**Fall**		6 (11.5%)	
**Motor vehicle accident**		27 (51.9%)	
**Sport**		7 (13.5%)	
**Other**		10 (19.2%)	
**Unknown**		1 (1.9%)	

Means and standard deviations are presented unless noted otherwise for days since injury and injury mechanism.

a–indicates t-test used for testing group differences.

b–indicates Mann Whitney U used for testing group differences.

Bolded rows indicate a significant group difference (P < 0.05).

DHI, Dizziness Handicap Inventory; NSI, Neurobehavioral Symptom Inventory; ANAM, Automated Neuropsychological Assessment Metrics; SOT, Sensory Organization Test.

### Protocol

Participants attended two data collection sessions where demographic, patient-reported questionnaires and clinical tests of vestibular and ocular motor function were collected first, and balance and neurocognitive function data were acquired approximately 1 week later. Patient-reported evaluation of mTBI-related symptoms was measured with the Neurobehavioral Symptom Inventory (NSI) (32, 33). The NSI includes 22 items, and each item is rated from 0 (none) to 4 (very severe). The NSI is comprised of a total score and this self-report questionnaire has been validated previously and has good internal consistency and stability (34, 35). The Dizziness Handicap Inventory (DHI) was used to assess the functional, emotional, and physical effects of dizziness using a 25-item questionnaire (36, 37). Participants rated each item according to the perceived handicap caused by their dizziness using 0 (no handicap), 2 (sometimes), or 4 (yes). Psychiatric effects of mTBI were quantified with Beck's Depression Inventory (BDI) and the Post-Traumatic Stress Disorder Checklist (PCL for DSM-IV (38, 39).

After completing demographic and patient-reported questionnaires, vestibular and ocular motor tests were performed that included bithermal bilateral caloric tests, video head impulse tests of horizontal canal function, cervical and ocular vestibular evoked myogenic potential tests of otolith function, Dix-Hallpike test for benign paroxysmal positional vertigo, horizontal random saccade tests, and horizontal smooth pursuit function tests. Results from these tests were previously reported (7). Neurocognitive function and balance performance were evaluated on a separate day. Neurocognitive function was evaluated with the Automated Neuropsychological Assessment Metrics (ANAM) test in a quiet environment on a computer (40). The ANAM composite score provided a neurocognitive demographic outcome to describe our groups. Balance performance was evaluated with the SOT and the CSMI test using a modified SMART EquiTest CRS Balance Manager system (Natus Medical Inc, Seattle WA). Participants completed 3 20-s trials of all 6 conditions of the SOT following recommended clinical test procedures. The CSMI test was performed using 12 repeated 20-s duration cycles of low-amplitude (2° or 4° peak-to-peak), wide-bandwidth pseudorandom stimuli that rotated the support surface and/or visual surround (6, 20). Four CSMI test conditions were performed including (1) surface-tilt with eyes closed – SS/EC, (2) surface tilt with eyes open viewing a fixed visual surround – SS/EO, (3) visual surround tilt with eyes open with stance on a level surface – VS/EO, and (4) combined surface-tilt and visual-tilt with eyes open – SS+VS/EO. Participants performed a warmup trial and then 8 test trials (4 conditions x 2 amplitudes with each lasting approximately 4 min) in one session with test trials presented in a randomized order and interspersed with planned rest breaks. The participants' anteroposterior (AP) body CoM displacement was derived from phaseless 2^nd^ order lowpass filtering (cutoff frequency 0.469 Hz) of AP CoP and AP CoM body sway angle was calculated using CoM height measures [see Peterka et al. (20) for details]. Due to a greater number of falls and incomplete CSMI tests performed using the 4° stimuli among mTBI participants, only results from the four CSMI tests performed using 2° stimuli are reported in this study.

### SOT analysis

The SOT analysis software provided SOT equilibrium scores for each trial with values ranging from 0, indicating an inability to maintain stance until the end of the 20-s trial, to 100 indicating perfect stability. A weighted combination of equilibrium scores was used to compute the SOT composite score that provided a conventional, objective measure of balance performance (41). The SOT equilibrium scores from HC and mTBI participants were further analyzed using the criteria developed by Cevette et al. (42) to classify subjects into “Normal,” “Vestibular Dysfunction,” or “Aphysiologic” patterns of SOT performance.

### CSMI model-based analysis

**Figure 1:**
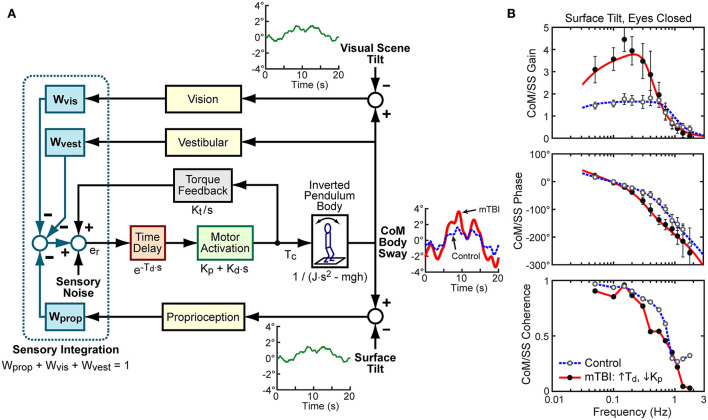
**(A)** Block diagram of Central Sensorimotor Integration (CSMI) feedback control model of the balance system used to identify functionally relevant balance control parameters of individual subjects. External stimuli, that include toe-up/toe-down rotational tilts of the stance surface and/or forward/backward tilts of the visual scene, evoke anterior-posterior (AP) body center-of-mass (CoM) sway responses represented by angular changes with respect to an upright vertical orientation of a body represented as an inverted pendulum. An internal “Sensory Noise” source is assumed to account for the observed variability of CoM sway. Example cycle-average stimulus-evoked sways are shown from two subjects from an eyes-closed, surface-tilt trial. The control subject's average sway (thin blue dotted line) was close to the average across all control subjects. The mTBI subject (thick red line) was from a subset of mTBI subjects with identified CSMI parameters with particularly long time delays, *T*_*d*_, and low motor activation stiffness, *K*_*p*_, values. **(B)** Examples of frequency domain analyses that calculated the control and mTBI subjects experimental Frequency Response Function (FRF) expressed as gain (ratio of CoM response amplitude to stimulus amplitude) and phase (normalized timing of response relative to the stimulus) as a function of stimulus frequency. The frequency domain analyses also provided Coherence Functions that characterized the signal-to-noise characteristics of the stimulus-response data with higher coherence values indicating better signal-to-noise. The experimental FRFs for the two example subjects are shown as filled (mTBI) or open (control) points with 95% confidence error bars. The solid and dashed lines in the gain and phase plots show the model-predicted FRFs after model parameters (time delay, *T*_*d*_, stiffness, *K*_*p*_, damping, *K*_*d*_, torque feedback, *K*_*t*_, and sensory weights, *W*'s) were optimally adjusted to account for the experimental FRFs.

The primary outcome measures of CSMI are parameters derived from a mathematical model-based interpretation of the CoM sway angle evoked by the pseudorandom stimuli (Figure 1A). A detailed description of how these parameters were calculated is explained elsewhere (6, 20). The mathematical model represents the body as an inverted pendulum that rotates about the ankle joint and is stabilized by a corrective ankle torque generated as a function of body motion information derived from sensory systems. The sensory sources include proprioception, sensing ankle joint motion, vision, sensing body sway relative to the visual scene, and vestibular, sensing body motion in space. An internal estimate of body orientation is formed using a weighted combination of motion cues from these three sensory sources (*W*_*prop*_, *W*_*vis*_, and *W*_*vest*_ are the parameters representing the proprioceptive, visual, and vestibular weights, respectively). The weights quantify the relative contribution of each sensory system such that the weights contributing to balance in a given condition sum to one. This sensory-weighted internal estimate of body orientation is modified by feedback that is a function of the mathematical integration of a sensory-derived measure of overall corrective torque applied at the ankle joint multiplied by a gain factor, *K*_*t*_, to give a final internal estimate of body orientation.

The balance control system cannot act instantaneously to generate corrective torque as a function of sensory information since there are delays in the system that are represented by the time delay parameter, *T*_*d*_. The delays include sensory transduction, afferent and efferent neural transmission, central sensorimotor processing, and muscle activation delays.

A motor activation component of the model includes a “stiffness” parameter, *K*_*p*_, and a “damping” parameter, *K*_*d*_, that generate the ankle torque as a function of the time-delayed internal body orientation estimate. *K*_*p*_ determines a component of the corrective torque proportional to the angular position of the internal motion estimate and *K*_*d*_ determines a component of the corrective torque proportional to the angular velocity. Finally, since *K*_*p*_ and *K*_*d*_ were previously shown to scale with the body mechanics, across-subject comparisons normalized *K*_*p*_ and *K*_*d*_ values by dividing by the product *mgh* where *m* is the subject mass (not including the feet), *g* is the gravity constant, and *h* is the subject's CoM height above the ankle joint (20). Ankle torque moves the inverted pendulum body with body mechanics determined by *mgh* and the moment of inertia, *J*, of the body about the ankle joint.

For each trial, frequency domain methods were used to calculate an experimental frequency response function (FRF) which is the ratio of the across-cycle average Fourier transform of CoM sway angle to the across-cycle average Fourier transform of the stimulus waveform (43). An FRF consists of complex numerical values as a function of frequency that are typically represented as FRF gain values (the magnitude of the complex values) and FRF phase values representing the timing of the CoM response relative to the stimulus (Figure 1B). The experimental FRF was compared to the model-predicted FRF and the model parameters were adjusted using the Matlab “fmincon” function (Matlab version R2019b and Matlab Optimization Toolbox; The MathWorks Inc., Natick Massachusetts) to minimize the error between the experimental and model-predicted FRF [see Peterka et al. (20)]. This analysis was performed for each subject's FRF in all four conditions. For illustrative purposes, Figure 1B shows two examples of individual experimental FRFs, and the optimal model FRF fits to the experimental FRFs from CSMI tests performed in the SS/EC condition. One FRF is from an HC participant whose fit parameters, including the vestibular weight *W*_*vest*_ = 0.44, were close to the mean values across all HC participants. The other FRF is from an mTBI participant who was among a subset of mTBI participants that had noticeably lengthened *T*_*d*_ and reduced *K*_*p*_ values but had a vestibular contribution to balance (*W*_*vest*_ = 0.47) that was close to the mean of HC participants.

Sensory weights derived directly from the model fits depended on the test condition. For SS/EC, SS/EO, VS/EO, and SS+VS/EO the model fits yielded the values *W*_*prop*_, *W*_*prop*_, *W*_*vis*_, and *W*_*prop*_+ *W*_*vis*_, respectively (see Supplementary materials). The sensory integration constraint of the model (sum of the contributing weights equals one) means that the four conditions also allows the calculation of *W*_*vest*_, *W*_*vis*_+ *W*_*vest*_, *W*_*prop*_+ *W*_*vest*_, and *W*_*vest*_, respectively.

In addition to the model-based analysis, we also calculated (1) the root mean square (RMS) value of stimulus-evoked CoM sway from the zero-meaned, cycle-averaged, CoM sway angle data from the final 11 cycles of the pseudorandom stimulus, (2) the RMS value of the “remnant sway” which is the square root of the sway variance that is not accounted for by the mean sway response to the stimulus (43, 44), and (3) an estimate of the RMS value of the “internal sensory noise” using the assumption that internal sensory noise is the source of variability that accounts for the measured remnant sway. See the Supplementary materials for details about the calculation of remnant sway and internal sensory noise.

### Statistical analysis

We evaluated group demographic differences on height, mass, NSI, DHI, BDI, PCL, ANAM, and SOT outcomes with independent Welch's *t*-tests, and Mann-Whitney *U* tests where appropriate. All CSMI outcome parameters were determined to be normally distributed, except for *K*_*t*_, after inspecting data within each group using Shapiro-Wilk tests, histograms, and q-q plots. The *K*_*t*_ parameter was log base 10 transformed to satisfy normality assumptions. Several independent Welch's *t*-tests compared group differences (HC vs. mTBI) on CSMI sensory weights, time delay, torque feedback, normalized stiffness, and damping parameters, and RMS values of CoM sway, remnant CoM sway, and internal sensory noise across the 4 CSMI conditions. All significance values were corrected for multiple comparisons using a Benjamini-Hochberg false discovery rate correction (45). Cohen's d effect sizes (Cohen's d_s_) were used to quantify the magnitude of standardized group differences (HC vs. mTBI) on CSMI model-based and RMS sway outcomes (46). We quantified the number of mTBI participants with abnormally long time delay, low normalized stiffness, and high internal sensory noise outcomes by using 90^th^, 10^th^, and 90^th^ percentile cutoffs, respectively, determined from all HC data (7). A Chi-Squared test associated the proportion of normal and abnormal combinations of time delays, normalized stiffness, and internal sensory noise with health status (HC or mTBI). Lastly, we used linear regression analysis to characterize the relationships between sensory weights, time delay, and normalized stiffness to assess the contribution of these parameters to the sensitivity to balance perturbations quantified by the RMS values of CoM stimulus-evoked sway. Independent Welch's *t*-tests and linear regression analyses were performed in MATLAB (r2019b). Chi-Squared analyses were carried out using SAS version 9.4.

## Results

The HC group and mTBI group were well matched for age, height, mass, and sex (Table 1). Participants with chronic mTBI were a median 398 days from their injury and over 50% of mTBI participants got their injury from a motor vehicle accident (Table 1). While all mTBI participants were > 3 months following their mTBI, they still had significantly higher NSI, DHI, BDI, and PCL total scores compared to the HC group (Table 1). The mTBI group had significantly worse ANAM composite scores relative to the HC group (Table 1). Additionally, the chronic mTBI group had significantly reduced balance performance, determined by a lower SOT composite score, compared with the HC group (Table 1).

Below we first describe various sway measures to provide an overview of the magnitude of sway evoked in the four CSMI stimulus conditions and the variability of that sway about the mean. The CSMI model parameters are then cataloged with significant differences between mTBI and HC measures identified. The relationship between the stimulus-evoked sway and the identified model parameters is described to facilitate an understanding that evoked sway depends on both sensory integration and motor activation properties. The unexpected presence of long time delays in mTBI subjects and their relationship with motor activation stiffness is characterized. Finally, the relationships between CSMI and SOT measures of balance are evaluated with particular regard to whether CSMI results can give insight into the causes of the Aphysiologic SOT performance pattern.

### CSMI stimulus-evoked CoM sway, remnant sway, and internal sensory noise

**Figure 2:**
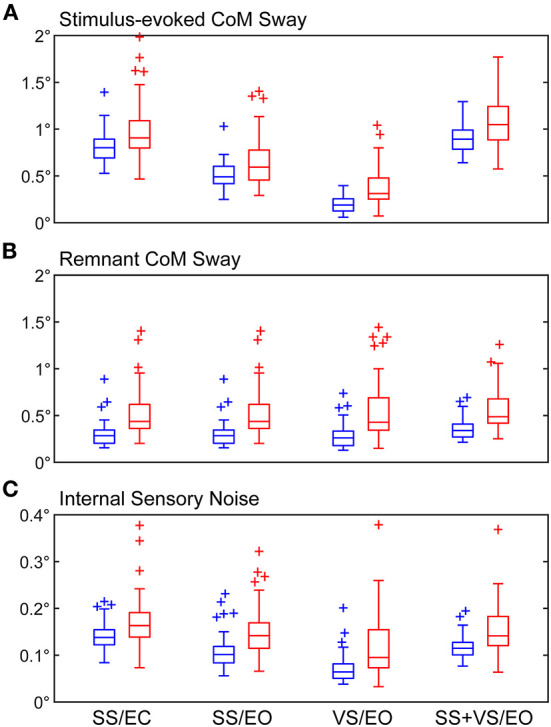
Boxplots of RMS values of **(A)** stimulus-evoked CoM sway, **(B)** remnant CoM sway, and **(C)** internal sensory noise. Results are shown for both healthy control (blue) and mTBI (red) subjects for the four CSMI test conditions. Central box shows median (center line) with upper and lower quartiles defined by 0.75 quantile and 0.25 quantile values, respectively. Outliers (+) are defined as values that are >1.5 times the interquartile range. Whiskers extend to maximum and minimum values that are not considered outliers.

Summaries of stimulus-evoked sway, remnant sway, and internal sensory noise measures are provided in Table 2 and Figure 2 and discussed below.

**Table 2:** RMS values of stimulus-evoked CoM sway, remnant CoM sway, and internal sensory noise for healthy controls (HC) and chronic mild traumatic brain injury (mTBI) groups.

**CSMI condition and measure**	**HC**	**mTBI**	**Adjusted *P*-value**	**Cohen's d_s_**
SS/EC				
N completing condition	58	49		
**Stimulus-evoked CoM Sway**	**0.810 (0.162)**	**0.973 (0.307)**	**0.0013**	**0.681**
**Remnant CoM Sway**	**0.423 (0.130)**	**0.628 (0.393)**	**0.0009**	**0.726**
**Internal Sensory Noise**	**0.142 (0.030)**	**0.183 (0.105)**	**0.0109**	**0.551**
**SS/EO**				
N completing condition	58	50		
**Stimulus-evoked CoM Sway**	**0.494 (0.140)**	**0.644 (0.256)**	**0.0004**	**0.742**
**Remnant CoM Sway**	**0.301 (0.129)**	**0.538 (0.341)**	**<0.0001**	**0.946**
**Internal Sensory Noise**	**0.107 (0.036)**	**0.160 (0.095)**	**0.0005**	**0.800**
**VS/EO**				
N completing condition	55	46		
**Stimulus-evoked CoM Sway**	**0.188 (0.088)**	**0.360 (0.204)**	**<0.0001**	**1.13**
**Remnant CoM Sway**	**0.280 (0.126)**	**0.555 (0.341)**	**<0.0001**	**1.11**
**Internal Sensory Noise**	**0.070 (0.029)**	**0.119 (0.068)**	**0.0141**	**0.968**
**SS+VS/EO**				
N completing condition	58	49		
**Stimulus-evoked CoM Sway**	**0.892 (0.159)**	**1.086 (0.289)**	**0.0001**	**0.852**
**Remnant CoM Sway**	**0.356(0.102)**	**0.578 (0.300)**	**<0.0001**	**1.03**
**Internal Sensory Noise**	**0.118 (0.023)**	**0.161 (0.074)**	**0.0002**	**0.814**

Outcomes presented as means and standard deviations.

All sway and noise measures have units of degrees.

Bolded outcomes indicate a significant group difference (Benjamini-Hochberg adjusted P < 0.05).

Abbreviations: N, number of participants; EC, eyes closed; EO, eyes open; SS, support surface stimulus; VS, visual surround stimulus; RMS, root mean square; CoM, center of mass.

For both HC and mTBI subjects, the RMS values of stimulus-evoked CoM sway were larger in the SS/EC and SS+VS/EO than in the SS/EO and VS/EO conditions (Figure 2A). In the SS/EC and SS+VS/EO conditions, the vestibular system was the only source of accurate orientation cues. When both visual and vestibular orientation cues were accurate in the SS/EO condition stimulus-evoked sways were reduced. The lowest stimulus-evoked sway was seen in the VS/EO condition where both proprioception and vestibular orientation cues were accurate.

The mTBI group had significantly larger RMS values of stimulus-evoked CoM sway relative to the HC group for all CSMI conditions indicating that, on average, the balance of mTBI subjects was disturbed to a greater extent than HC subjects by rotational perturbations applied to the stance surface and/or visual scene. The largest difference in mean values of RMS CoM sway was observed for the VS/EO condition (92% larger for the mTBI group, Cohen's d_s_ = 1.13; Table 2).

The mTBI group also had significantly larger mean RMS values of remnant sway (the CoM sway variance that is not accounted for by the mean CoM sway response to the stimulus) relative to the HC group for all CSMI conditions with the largest difference (98% larger for the mTBI group) also observed for the VS/EO condition (Cohen's d_s_ = 1.11; Figure 2B). A similar trend emerged for the RMS value of internal sensory noise (Figure 2C); the mTBI group had significantly larger RMS values of internal sensory noise relative to the HC group for all CSMI conditions. The largest difference (70% larger for the mTBI group) was observed during the VS/EO condition (Cohen's d_s_ = 0.968).

### CSMI parameters

Summaries of CSMI parameter values and statistical comparisons between results from HC and mTBI subjects are provided in Table 3 and discussed below.

**Table 3:** Central Sensorimotor Integration (CSMI) test model-derived parameters for healthy controls (HC) and chronic mild traumatic brain injury (mTBI) groups.

**CSMI condition and parameter**	**HC**	**mTBI**	**Adjusted *P*-value**	**Cohen's d_s_**
SS/EC				
N completing condition	58	49		
Proprioceptive weight	0.509 (0.085)	0.522 (0.073)	0.3953	0.163
Vestibular weight	0.491 (0.085)	0.478 (0.073)	0.3953	0.163
**Time delay (ms)**	**150 (14.0)**	**165 (24.0)**	**0.0001**	**0.780**
Torque feedback (rad/Nms)^a^	−3.966 (0.186)	−3.99 (0.224)	0.7595	0.118
**Normalized stiffness**	**1.505 (0.129)**	**1.414 (0.149)**	**0.0012**	**0.657**
**Normalized damping**	**0.531 (0.069)**	**0.493 (0.058)**	**0.0025**	**0.592**
SS/EO				
N completing condition	58	50		
Proprioceptive weight	0.298 (0.051)	0.314 (0.059)	0.1354	0.292
Vestibular + visual weight	0.702 (0.051)	0.686 (0.059)	0.1354	0.292
**Time delay (ms)**	**132 (21.0)**	**145 (29.0)**	**0.0076**	**0.520**
Torque feedback (rad/Nms)^a^	−4.015 (0.205)	−4.068 (0.296)	0.2887	0.211
**Normalized stiffness**	**1.578 (0.231)**	**1.432 (0.174)**	**0.0003**	**0.707**
**Normalized damping**	**0.527 (0.074)**	**0.469 (0.077)**	**0.0001**	**0.769**
VS/EO				
N completing condition	55	46		
**Visual weight**	**0.108 (0.045)**	**0.151 (0.059)**	**0.0001**	**0.830**
**Proprioceptive** **+** **vestibular weight**	**0.892 (0.045)**	**0.849 (0.059)**	**0.0001**	**0.830**
**Time delay (ms)**	**200 (21.0)**	**216 (27.0)**	**0.0015**	**0.669**
**Torque feedback (rad/Nms)**^**a**^	**−4.770 (1.496)**	**−4.233 (0.661)**	**0.0189**	**0.451**
Normalized stiffness	1.267 (0.101)	1.222 (0.173)	0.1231	0.325
Normalized damping	0.502 (0.056)	0.482 (0.055)	0.0679	0.360
**SS+VS/EO**				
N completing condition	58	49		
Proprioceptive + visual weight	0.552 (0.064)	0.567 (0.068)	0.2420	0.228
Vestibular weight	0.448 (0.064)	0.433 (0.068)	0.2420	0.228
**Time delay (ms)**	**140.0 (18.0)**	**165 (29.0)**	**<** **0.0001**	**1.06**
Torque feedback (rad/Nms)^a^	−3.986 (0.193)	−3.973 (0.203)	0.7386	0.066
**Normalized stiffness**	**1.488 (0.134)**	**1.375 (0.136)**	**<** **0.0001**	**0.838**
**Normalized damping**	**0.504 (0.078)**	**0.465 (0.055)**	**0.0034**	**0.570**

Outcomes presented as means and standard deviations.

a–Mean and standard deviation of torque feedback based on log10 of parameter values.

Bolded outcomes indicate a significant group difference (Benjamini-Hochberg adjusted P < 0.05).

N, number of participants; EC, eyes closed; EO, eyes open; SS, support surface stimulus; VS, visual surround stimulus; ms, milliseconds; rad, radians; Nms, Newton meter second.

#### Sensory weights

People with mTBI relied significantly more on visual cues (40% larger visual weight, Cohen's d_s_ = 0.830) during the VS/EO condition compared to the healthy controls. There were no significant differences in sensory weighting for SS/EC, SS/EO, and SS+VS/EO conditions. Specifically, mTBI participants had similar reliance (only 3% less) on vestibular cues relative to HC participants for SS/EC and SS+VS/EO conditions. Additionally, both mTBI and HC participants had similar reliance (mTBI only 5% more) on proprioceptive cues in the SS/EO condition.

#### Time delay

The mTBI participants had significantly longer time delays compared to HC participants for all CSMI conditions. Specifically, mean time delays for the mTBI participants were 10%, 10%, 8%, and 18% longer than the HC participants' mean time delays for SS/EC, SS/EO, VS/EO, and SS+VS/EO conditions, respectively (Cohen's d_s_ effect sizes ranging from 0.52 to 1.06; Table 3).

#### Motor activation – normalized stiffness and damping

Both motor activation components were significantly reduced in the mTBI group compared to the HC group for SS/EC, SS/EO, and SS+VS/EO conditions. Specifically, the mean normalized stiffness parameters were 6, 9, and 8% reduced in the mTBI participants relative to the HC participants for SS/EC, SS/EO, and SS+VS/EO conditions, respectively. Similarly, mTBI participants' mean normalized damping was reduced by 7, 11, and 7% relative to the HC participants. For the VS/EO condition, there was no significant difference in motor activation parameters between the mTBI group and the HC group with 4% reductions in mean normalized stiffness and damping for mTBI participants relative to HC participants (see Table 3 for Cohen's d_s_ effect sizes).

#### Torque feedback

The mean torque feedback for mTBI participants was significantly larger (11%, Cohen's d_s_ = 0.451) relative to the HC participants during the VS/EO condition. In all other test conditions, there were no other significant differences (< 1%, Cohen's d_s_ < 0.211) between mTBI and HC participants on the torque feedback parameter. The torque feedback parameter determines the sensitivity to balance perturbations at lower frequencies (below about 0.1 Hz) with larger values associated with reduced sensitivity.

### Relations among CSMI sway measures and CSMI parameters

Across all test conditions, mTBI subjects showed, on average, greater balance disturbances (i.e., greater stimulus-evoked sway indicating increased sensitivity to the stimulus) than the HC subjects. CSMI parameters that characterize mechanisms of the balance control system were used to investigate the causes of the greater sensitivity to balance disturbances in mTBI compared to HC subjects.

**Figure 3:**
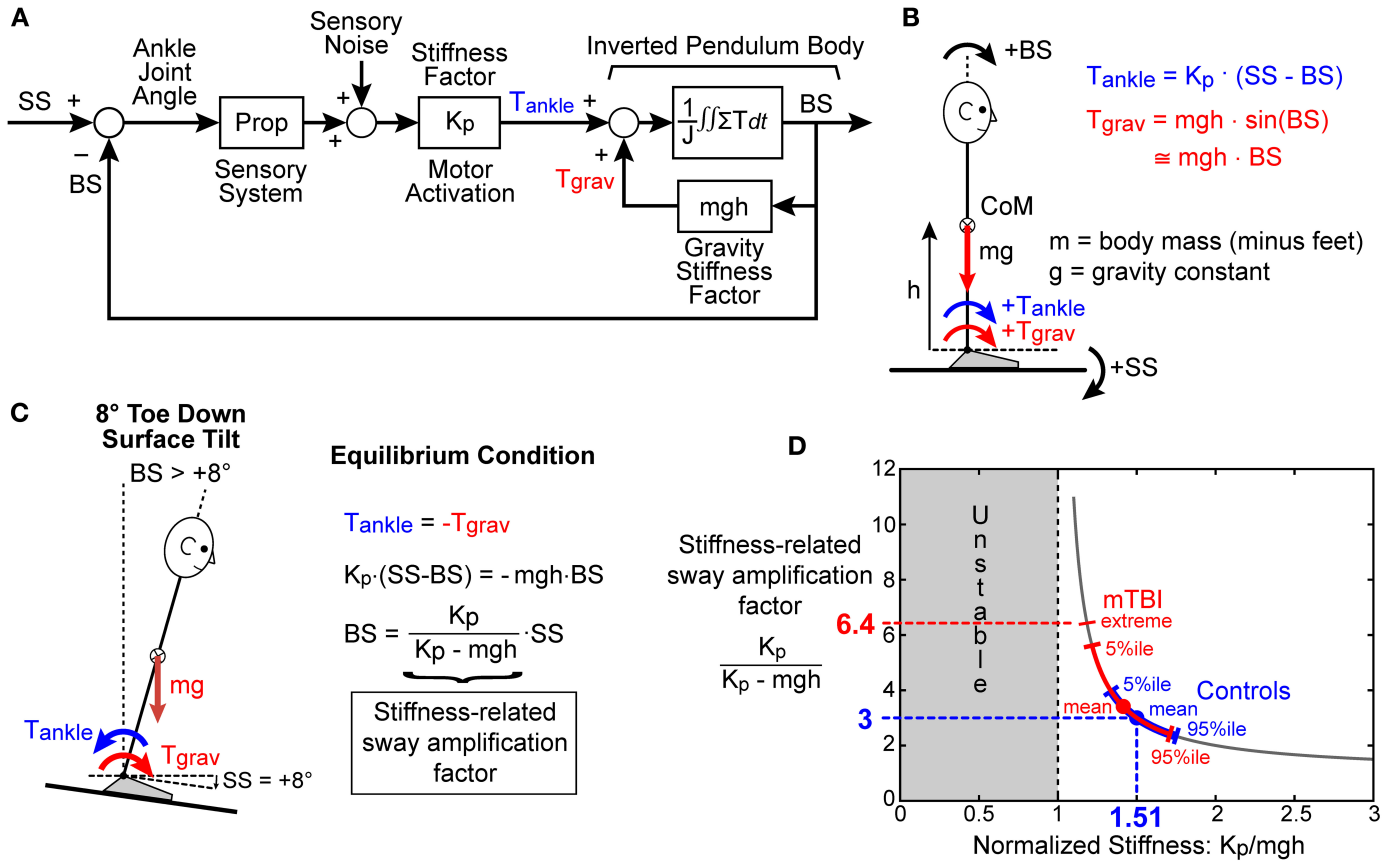
Simplified model used to explain the potential for the motor activation stiffness factor to determine the sensitivity to external and internal balance perturbations. **(A)** A greatly simplified feedback control model where proprioception accurately senses ankle joint angle, which is the difference between the stance surface (*SS*) angle and body sway (*BS*) angle, and then generates ankle torque, *T*_*ankle*_, in proportion to this difference with the proportional stiffness factor *K*_*p*_. Lean of the body away from vertical generates a destabilizing torque due to gravity, *T*_*grav*_, proportional to *BS* with a “gravity stiffness” proportional factor *mgh* (mass x gravity constant x height of body center-of-mass). **(B)** The various biomechanical factors and the positive directions of *SS, BS, T*_*ankle*_, and *T*_*grav*_ are defined. **(C)** When the stance surface is rotated to a non-horizontal orientation, the balance control system will cause the body to move to a new equilibrium orientation where *T*_*ankle*_ is equal in magnitude but opposite in direction to *T*_*grav*_. The *BS* equilibrium angle is proportional to *SS* but is always greater than *SS* with the proportional factor of *K*_*p*_/(*K*_*p*_ - *mgh*) defined as the “Stiffness-related sway amplification factor.” **(D)** Graph showing the hyperbolic relationship between *K*_*p*_/(*K*_*p*_ - *mgh*) and normalized stiffness factor *K*_*p*_/*mgh*. The mean values of *K*_*p*_/*mgh* and the range of values for control (blue) and mTBI subjects (red) measured in the SS/EC condition are overlaid on the hyperbolic curve and show generally lower *K*_*p*_/*mgh* and higher *K*_*p*_/(*K*_*p*_ - *mgh*) values for mTBI subjects resulting in increased sensitivity to balance perturbations.

Increased sensitivity to balance disturbances could result from increased reliance on sensory cues for balance, with increased reliance represented by CMSI sensory weights. A less obvious mechanism affecting response sensitivity is altered control of the corrective ankle torque generated per unit of body sway. This altered control is represented by the motor activation mechanism that converts sensory-detected body motion to corrective torque and is represented by the stiffness (*K*_*p*_) and damping (*K*_*d*_) parameters of the CSMI model (Figure 1A). To illustrate how motor activation, and in particular the stiffness parameter, affects responsiveness to perturbations, Figure 3 uses a greatly simplified balance control model, that includes only proprioception to encode body sway (Figure 3A), to show that reduced stiffness would be expected to increase sensitivity to a balance perturbation (Figure 3D). Specifically, the biomechanical factors and equations of motion defined in Figure 3B were used in Figure 3C to predict the equilibrium orientation of the body in response to a tilt of the stance surface. Importantly, the equilibrium tilt angle of the body is always greater than the surface tilt and is determined by a “stiffness-related sway amplification factor” equal to *K*_*p*_/(*K*_*p*_ - *mgh*). The graph in Figure 3D illustrates that as the normalized stiffness approaches a value of 1 the stiffness-related sway amplification factor greatly increases. That this analysis is relevant to the understanding of the increased sensitivity among mTBI subjects to balance perturbations is illustrated in Figure 3D, which shows the mean values and ranges of normalized stiffness values from the SS/EC condition for HC and mTBI subjects. The distribution of normalized stiffness values of mTBI subjects was generally shifted toward lower values than for HC subjects and included a distribution tail of lower values that resulted in rather large values of sway amplification factors for a subset of mTBI subjects.

**Figure 4:**
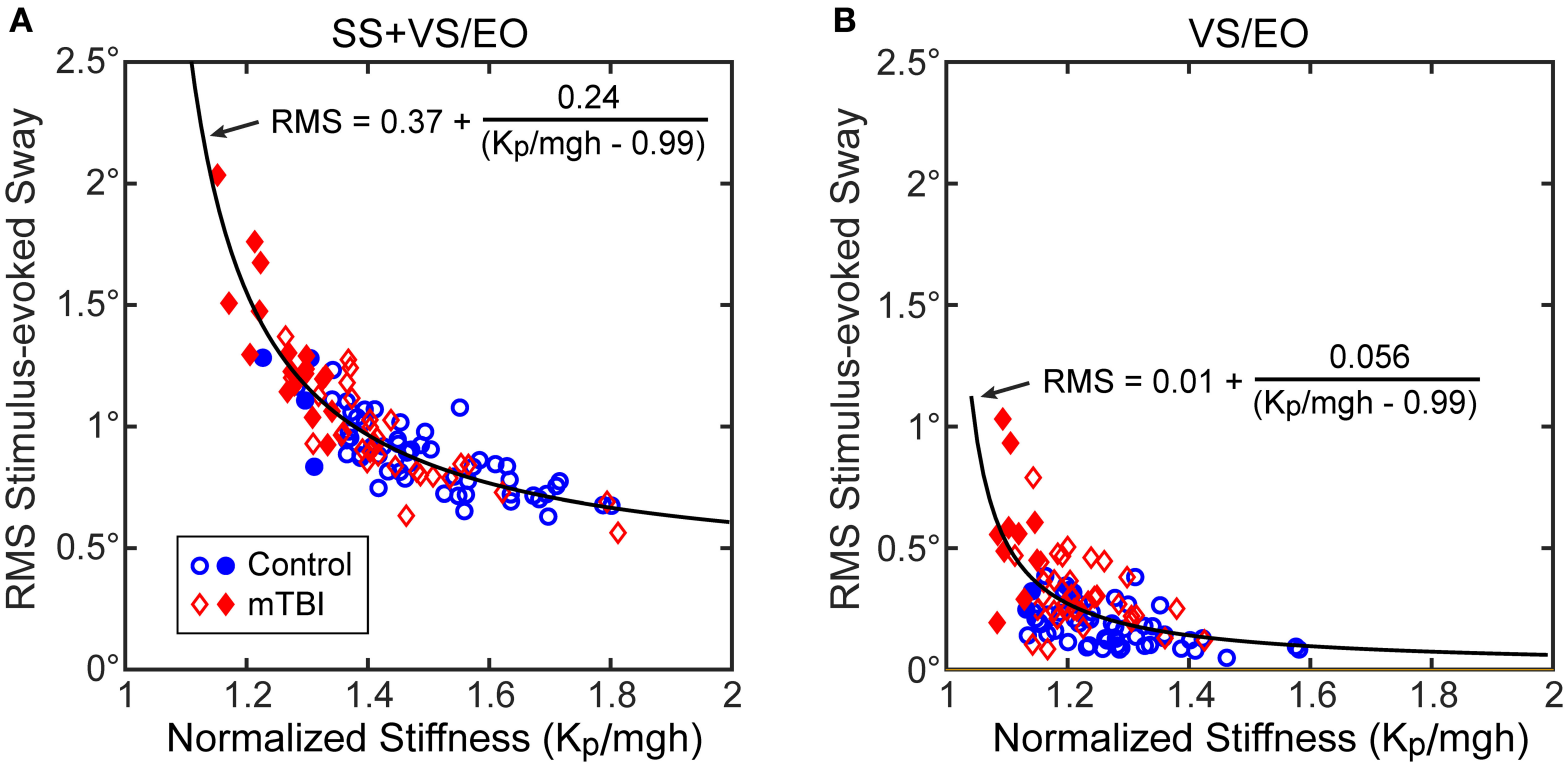
Relationships between the RMS value of stimulus-evoke CoM sway and normalized stiffness (*K*_*p*_/*mgh*) in **(A)** the SS+VS/EO test condition (simultaneous pseudorandom surface and visual tilt stimuli) and in **(B)** the VS/EO test condition (visual stimulus only). Data are from control subjects (blue circles) and mTBI subjects (red diamonds) with filled symbols indicating the 4 control and 19 mTBI subjects in the SS+VS/EO condition and the 2 control and 10 mTBI subjects in the VS/EO condition identified as having both low stiffness and long time delays based on 10^th^ percentile and 90^th^ percentile values of control subject data for normalized stiffness and time delay parameters, respectively (see Figure 7). Fits to the combined control and mTBI data of a hyperbolic curve are based on the expected relationship between stimulus-evoked sway and *K*_*p*_/*mgh* based on the prediction of the simplified balance control model shown in Figure 3.

Consistent with the above analysis, the RMS measure of stimulus-evoked sway showed a hyperbolic relationship with the normalized stiffness parameter derived from the CSMI analysis (Figure 4A results shown for the SS+VS/EO condition). Similar robust hyperbolic relationships were observed for the SS/EC and SS/EO conditions. However, while a hyperbolic relationship was still evident for the VS/EO condition (Figure 4B), it was less robust than in the other three conditions.

**Figure 5:**
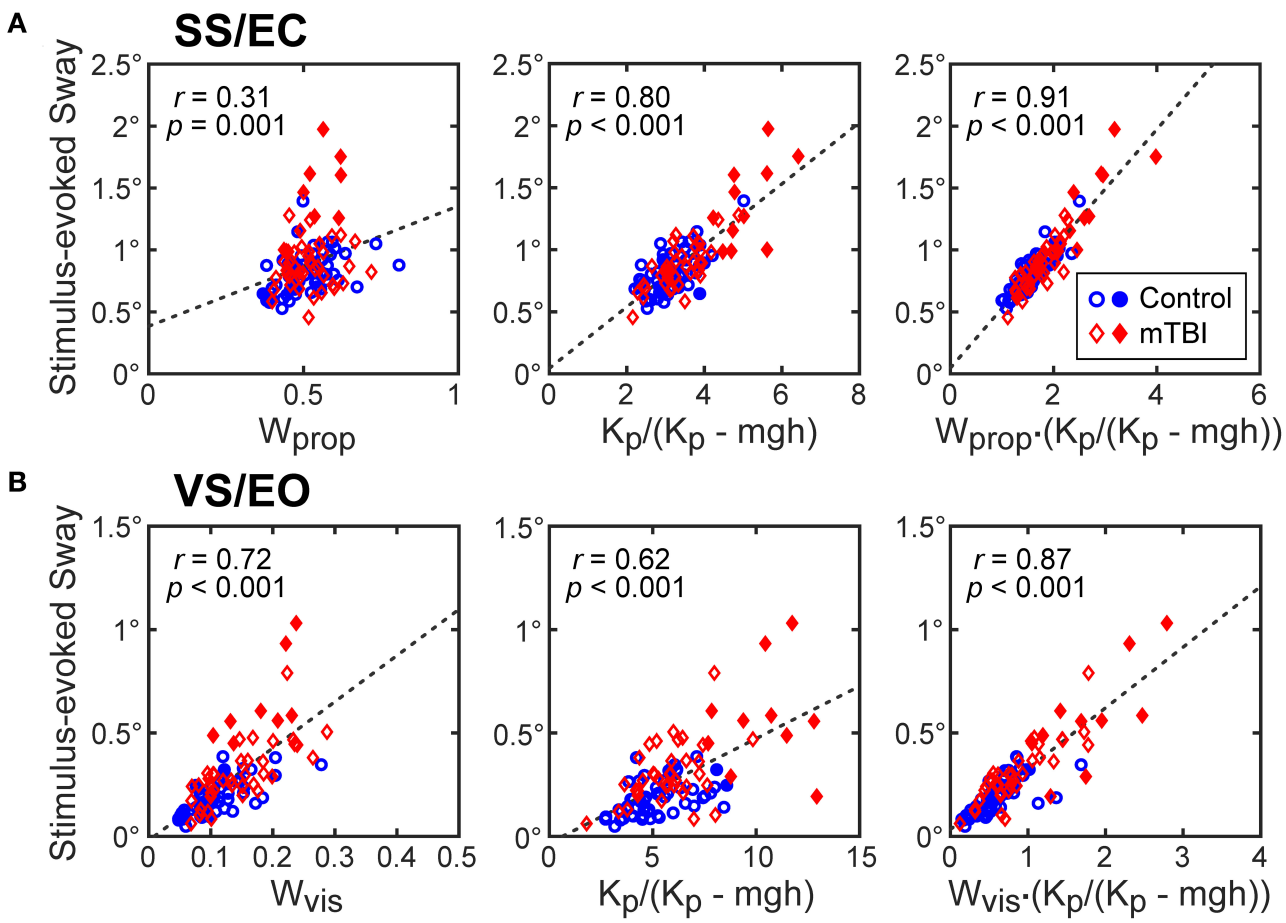
CSMI parameters that account for the variation in RMS values of stimulus-evoked CoM sway. Stimulus-evoked sway measures in control (blue dots) and mTBI (red diamonds) are plotted against CSMI sensory weight parameter, the stiffness-related sway amplification factor *K*_*p*_/(*K*_*p*_ - *mgh*), and the product of these two terms for data from **(A)** surface-tilt stimulus with eyes closed (SS/EC) trials and **(B)** visual-tilt stimulus with eye open (VS/EO) trials. Filled symbols are for the subset of subjects with particularly long time delay and low normalized stiffness values (see Figure 7).

The results shown in Figure 5 and Table 4 account for the differences among test conditions in the robustness of the hyperbolic relationship between normalized stiffness and stimulus-evoked sway. Specifically, CSMI model properties indicated that both the sensory weight and the stiffness parameter contributed to the sensitivity of the balance system to balance disturbances but the contribution of these two parameters varied between test conditions. To determine the contribution of each factor, the relations between the stimulus-evoked sway and (1) sensory weight, (2) the stiffness-related sway amplification factor *K*_*p*_/(*K*_*p*_ - *mgh*), and (3) the product of these two factors were determined. For the SS/EC condition, the correlation between stimulus-evoked sway and *K*_*p*_/(*K*_*p*_- *mgh*) was much higher than the correlation with the proprioceptive sensory weight (*W*_*prop*_; Figure 5A) with the variance accounted for by these linear relations summarized in Table 4. The relations for the SS/EO and SS+VS/EO conditions were similar to the SS/EC condition. In contrast, the results for the VS/EO condition showed that the visual sensory weight (*W*_*vis*_) accounted for more of the variance than the *K*_*p*_/(*K*_*p*_-*mgh*) factor (Figure 5B). This relatively low contribution of stiffness to account for stimulus-evoked sway in the VS/EO condition explained the less robust hyperbolic relation shown in Figure 4B compared to the other conditions. Finally, the fact that the product of the sensory weight times *K*_*p*_/(*K*_*p*_ - *mgh*) explained the largest amount of variance in all four conditions indicated that both factors contributed to the magnitude of stimulus-evoked sway and the combination of the two factors accounted for a large portion (76–90%) of the sway variance.

**Table 4:** Variance (r^2^) of RMS value of CoM stimulus-evoked sway explained by sensory weight factor alone, stiffness-related sway amplification factor alone, and product of the sensory weight with the stiffness-related sway amplification factor.

	**Sensory weight**	**Stiffness amplification factor**	**Sensory weighting x stiffness amplification factor**	**N (HC + mTBI)**
SS/EC	0.09	0.65	0.83	107
SS/EO	0.30	0.70	0.90	108
VS/EO	0.51	0.39	0.76	101
SS+VS/EO	0.02	0.81	0.83	107

Data from HC and mTBI subjects combined. N, number of participants; HC, healthy controls; mTBI, mild traumatic brain injury; EC, eyes closed; EO, eyes open; SS, support surface sway; VS, visual surround sway; RMS, root mean squared; CoM, center of mass.

**Figure 6:**
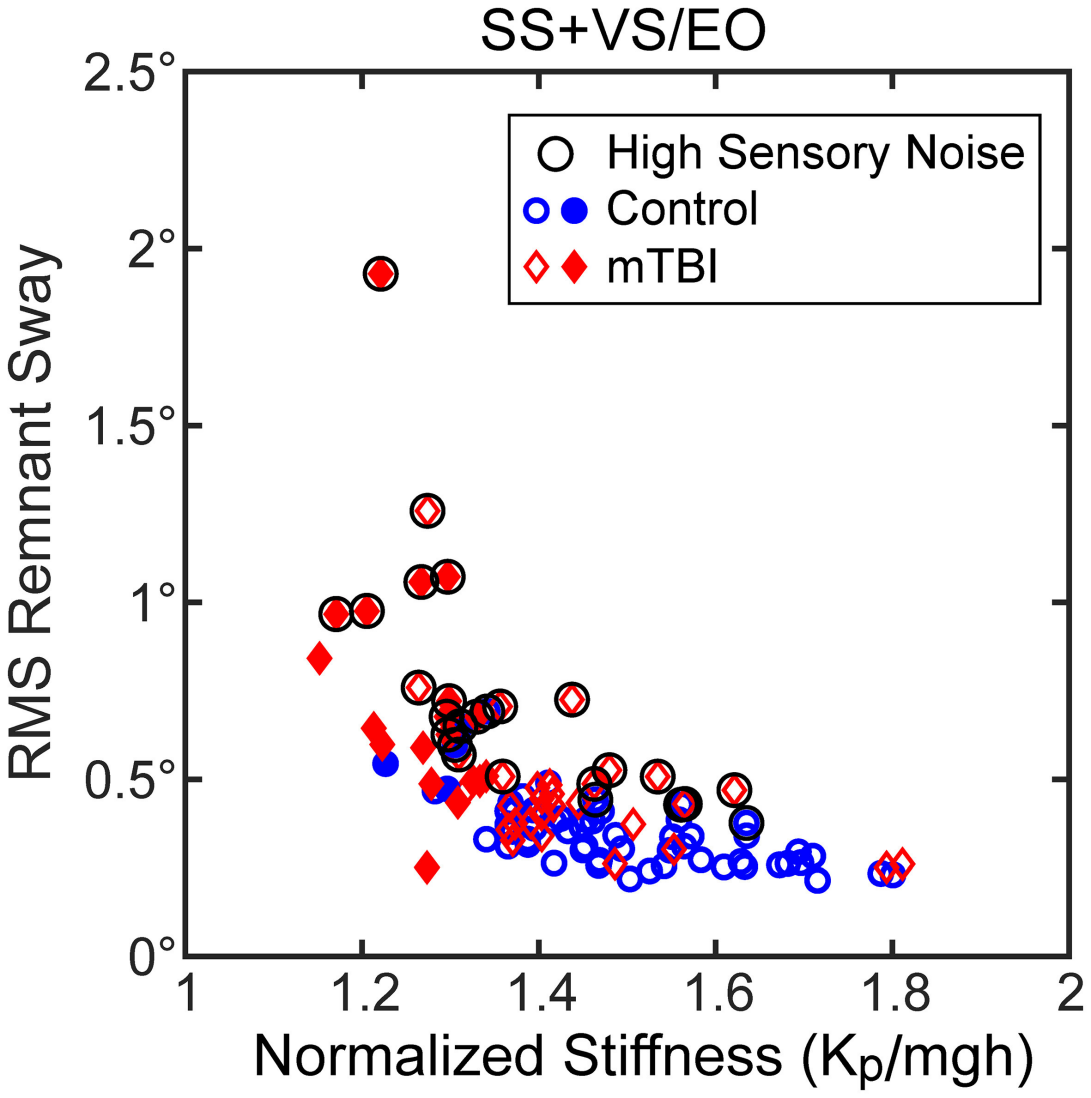
Relationships between RMS values of remnant CoM sway and normalized stiffness (*K*_*p*_/*mgh*) for the SS+VS/EO test condition (simultaneous pseudorandom surface and visual tilt stimuli). Data are from control subjects (blue circles) and mTBI subjects (red diamonds) with filled symbols indicating the 4 control and 19 mTBI subjects with both low stiffness and long time delays based on 10^th^ percentile and 90^th^ percentile values of control subject data for normalized stiffness and time delay parameters, respectively (see Figure 7). There is some evidence of a hyperbolic relationship between remnant sway and *K*_*p*_/*mgh* but there are outliers that show greater remnant sway than others at a given *K*_*p*_/*mgh* value. These outlying points are associated with subjects with greater internal sensory noise as indicated by the black circle symbol for subjects with internal sensory noise values greater than the 90^th^ percentile value for control subjects.

Assuming that remnant sway arises from an internal sensory noise source then the CSMI model predicts that the RMS value of remnant CoM sway should also be influenced by the stiffness parameter. The results shown in Figure 6 for the SS+VS/EO condition showed a hyperbolic relation between remnant sway amplitude and normalized stiffness that was similar to the results shown for the same condition in Figure 4A. However, there was more variability around a hyperbolic relationship. This increased variability was attributed to the fact that the magnitude of the internal sensory noise differed between subjects whereas stimulus-evoked sway for each subject in Figure 4A was in response to a stimulus of the same magnitude.

We used the assumption that the internal noise was attributable to sensory noise, based on the main finding of van der Kooij and Peterka (44) that balance system behavior was dominated by sensory noise properties. This assumption allowed us to estimate the magnitude of the internal sensory noise following the sensory integration process from the measured remnant sway and the dynamic balance characteristics identified by the CSMI model analysis. The estimates of internal sensory noise varied across subjects and were generally larger in mTBI subjects than in HC subjects (Figure 2C, Table 2). The circled points in Figure 6 indicate data from subjects whose amplitude of internal sensory noise was >90^th^ percentile value determined from HC data for this condition. These circled points corresponded to data that added considerable variability to the relationship between remnant sway and normalized stiffness.

### Time delay and stiffness relations

**Figure 7:**
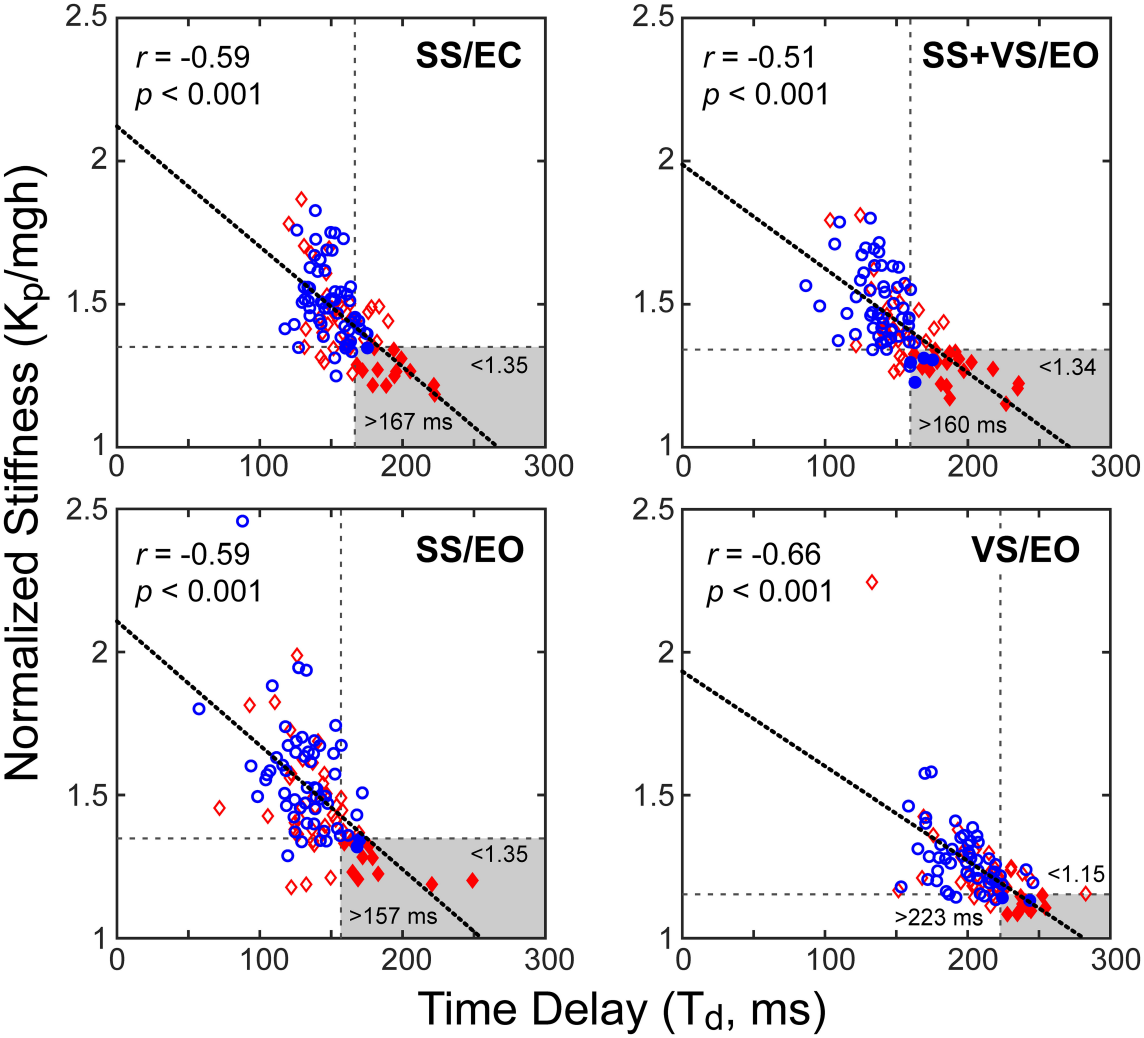
Correlations between normalized stiffness (motor activation stiffness parameter, *K*_*p*_, divided by *mgh* (mass x gravity constant x height of body center-of-mass) and time delay, *T*_*d*_, for the four test conditions for combined data from control subjects (blue circles) and mTBI subjects (red diamonds). Longer time delays were associated with lower normalized stiffness values (thick dotted lines: linear regression fits). Horizontal and vertical dashed lines show 10^th^ percentile and 90^th^ percentile values based on control subject data for normalized stiffness and time delay parameters, respectively. Gray areas show long time delay – low stiffness regions based on control-subject percentile cutoffs with filled symbols indicating subjects whose parameters were within these regions.

Results showed a consistent inverse relationship between the time delay and stiffness parameters in all test conditions (Figure 7). It was visually evident that mTBI subjects tended to have lower normalized stiffness and longer time delays than HC subjects and a subgroup of the mTBI subjects was largely separable from HC subjects based on a combination of low stiffness and longer time delays (stiffness below the 10^th^ percentile value and time delay above the 90^th^ percentile for HC subjects). A summary of the various categorizations based on these percentile cutoffs is given in Table 5 and includes statistical analysis that showed significant differences between the proportion of HC and mTBI subjects in the various categories. Across all four test conditions, an average of 28% of mTBI subjects were included in the long time delay – low stiffness subgroup compared to an average of 4% of the HC subjects.

**Table 5:** Number (N) and percentage (%) of healthy control (HC) and chronic mTBI participants with abnormal Central Sensorimotor Integration (CSMI) time delay, normalized stiffness, internal sensory noise, and combinations of these measures.

**CSMI condition and abnormal parameter**	**HC**	**mTBI**	**Adjusted *p*-value**
SS/EC			
Completed test	58	49	
**Time Delay**^**a**^	**6 (10%)**	**21 (43%)**	**0.002**
**Normalized Stiffness**^**b**^	**6 (10%)**	**20 (41%)**	**0.0009**
Internal sensory noise^c^	6 (10%)	12 (24%)	0.0773
**Both time delay and stiffness**	**1 (2%)**	**14 (29%)**	**0.001**
Time delay, stiffness, and sensory noise	1 (2%)	4 (8%)	0.1766
SS/EO			
Completed test	58	50	
**Time delay**^**a**^	**6 (10%)**	**15 (30%)**	**0.0188**
**Normalized stiffness**^**b**^	**6 (10%)**	**16 (32%)**	**0.012**
**Internal sensory noise**^**c**^	**6 (10%)**	**19 (38%)**	**0.0022**
**Both time delay and stiffness**	**2 (3%)**	**11 (22%)**	**0.0095**
Time delay, stiffness, and sensory noise	2 (3%)	7 (14%)	0.0821
VS/EO			
Completed test	55	46	
**Time delay**^**a**^	**5 (9%)**	**19 (41%)**	**0.0008**
**Normalized stiffness**^**b**^	**5 (9%)**	**14 (30%)**	**0.006**
**Internal sensory noise**^**c**^	**6 (11%)**	**23 (50%)**	**0.0007**
**Both time delay and stiffness**	**2 (4%)**	**10 (22%)**	**0.0153**
**Time delay, stiffness, and sensory noise**	**1 (2%)**	**7 (15%)**	**0.0292**
SS+VS/EO			
Completed test	58	49	
**Time delay**^**a**^	**6 (10%)**	**24 (49%)**	**0.0005**
**Normalized stiffness**^**b**^	**6 (10%)**	**23 (47%)**	**0.0004**
**Internal sensory noise**^**c**^	**6 (10%)**	**20 (41%)**	**0.0007**
**Both time delay and stiffness**	**4 (7%)**	**19 (39%)**	**0.0003**
**Time delay, stiffness, and sensory noise**	**2 (3%)**	**9 (18%)**	**0.027**

a–Percent abnormal based on time delay values that were >90^th^ percentile values derived from HC data.

b–Percent abnormal based on normalized stiffness values that were < 10^th^ percentile values derived from HC data.

c–Percent abnormal based on internal sensory noise values that were >90^th^ percentile values derived from HC data.

Bolded outcomes indicate a significant group difference (Benjamini-Hochberg adjusted P < 0.05).

N, number of participants; EC, eyes closed; EO, eyes open; SS, support surface stimulus; VS, visual surround stimulus.

### SOT and CSMI relationships

**Figure 8:**
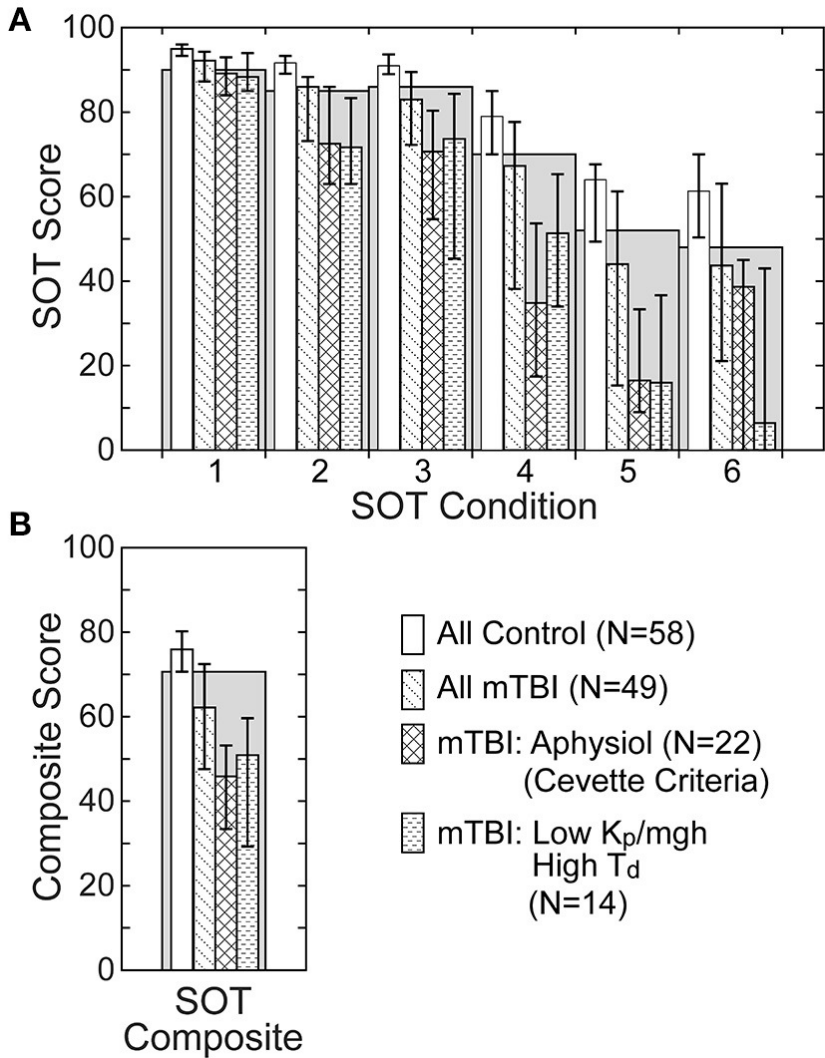
Sensory Organization Test (SOT) equilibrium scores **(A)** on the 6 SOT conditions and composite scores **(B)** for all healthy control subjects, all mTBI subjects, mTBI subjects classified as having an Aphysiologic pattern of SOT results using the criteria defined by Cevette et al. (42), and mTBI subjects with low normalized stiffness (*K*_*p*_/*mgh*) and long time delay (*T*_*d*_) parameters on the CSMI SS/EC test condition. Gray bars show the clinical 5^th^ percentile limit of SOT equilibrium scores for subjects aged 20–59 years (41). Bars for the various subject groups show median values with error bars indicating the range defined by 25^th^ and 75^th^ percentile values.

An overview of the SOT equilibrium scores in HC and mTBI subjects on the 6 SOT conditions (Figure 8A) and the SOT composite score (Figure 8B) is illustrated using data from the 58 HC and 49 mTBI subjects with results from both SOT and the SS/EC CSMI test. SOT group performance was generally poorer in mTBI than HC subjects across all test conditions with the median equilibrium score values for mTBI subjects being below the clinically defined 5^th^ percentile confidence limit on SOT conditions 3 to 6 and on the SOT composite score (41). Additionally, a large fraction (22 of 49, 45%) of the mTBI group were identified as having an “Aphysiologic” pattern using the criteria defined by Cevette et al. (42). As compared to the entire mTBI group, SOT measures showed consistently poorer performance in the Aphysiologic mTBI subjects with the median SOT composite score and SOT equilibrium scores below clinical norms.

Because the CSMI analysis identified a subgroup of the mTBI subjects who were largely separable from HC subjects, based on a combination of low stiffness and longer time delays (Figure 7, Table 5), it was of interest to examine SOT results for this subgroup and to identify the extent to which this subgroup overlapped with Aphysiologic mTBI subjects. For the SS/EC CSMI condition, 14 mTBI subjects were in the low stiffness - long time delay subgroup and 10 of these 14 (71%) were classified as Aphysiologic on SOT. Furthermore, SOT results from the low stiffness - long time delay subgroup showed poorer balance performance than the entire mTBI group with median equilibrium scores below clinical norms on all SOT conditions. Supplementary Table S1 shows the result of Cevette categorizations into Normal, Aphysiologic, and Vestibular Dysfunction patterns for HC and mTBI subjects in the 4 CSMI test conditions. Across all 4 CSMI test conditions, about 45% of the mTBI subjects were classified as Aphysiologic while <4% of HC were in this category. Supplementary Table S1 also shows the number of subjects who were in the low stiffness - long time delay subgroup and who were also classified as Aphysiologic by the Cevette criteria. Across all 4 CSMI test conditions for mTBI subjects, 50 to 71% of subjects in the low stiffness - long time delay subgroup were also classified as Aphysiologic.

Various other CSMI sway measures and model parameters also showed notable differences between mTBI subjects classified as Aphysiologic and “Not Aphysiologic” (i.e., subjects classified with Normal or with Vestibular Dysfunction SOT patterns), and in comparison to HC subjects. Supplementary Table S2 shows CSMI stimulus-evoked and remnant CoM sway measures and internal sensory noise for HCs and mTBI subjects in the Aphysiologic and Not Aphysiologic categories for all 4 CSMI test conditions. Across all CSMI test conditions, the CSMI sway and internal noise were smallest for HCs, larger for mTBI in the Not Aphysiologic group, and largest in the mTBI Aphysiologic group. All measures were statistically significantly larger in the Aphsyiologic group compared to both the Not Aphysiologic group and HCs. Additionally, all measures in the Not Aphysiologic group were significantly larger than in HCs in the VS/EO CSMI condition.

Supplementary Table S3 shows CMSI model parameters in the 4 CSMI test conditions for HCs and mTBI subjects in the Aphsiologic and Not Aphysiologic categories. For the SS/EC, SS/EO, and SS+VS/EO conditions the normalized stiffness and normalized damping were significantly lower and the time delay was significantly longer in the Aphysiologic mTBI group than in HCs. For the VS/EO condition, the visual weight and time delay were significantly larger in the both the Aphysiologic and Not Aphysiologic groups than in HCs.

## Discussion

Results showed significant differences between chronic mTBI subjects with self-reported balance complaints and HC subjects in the mean values of CoM body sway measures and in several parameters obtained from the CSMI model-based analysis. Across all four test conditions chronic mTBI subjects, on average, showed increased stimulus-evoked sway, increased remnant sway, and increased internal sensory noise. Across all test conditions, the mean CSMI time delay from mTBI subjects was significantly longer than in HC subjects. The mean value of other CSMI parameters also differed significantly from HC values but in a stimulus condition-dependent manner. Specifically, the mean visual weight was larger in mTBI than HC on the VS/EO condition while sensory weights identified on the other three conditions were not significantly different. Finally, mean values of motor activation parameters of stiffness and damping in mTBI subjects were significantly reduced relative to HC in the three test conditions that included surface-tilt stimulation (SS/EC, SS/EO, SS+VS/EO) but not in the VS/EO condition. Below we relate these results to other studies that have characterized balance function following mTBI using other methods and discuss the relationships between SOT and CSMI measures.

### Balance assessments using condition-dependent sway

It is important to distinguish between condition-dependent and stimulus-evoked sway. Subjects attempting to stand still continuously sway even when there is no external stimulus applied. The magnitude of this sway varies with test conditions with greater sway typically seen in conditions where the accuracy or availability of sensory information is diminished; for example, by eye closure, stance on foam, or sway-referencing of the stance surface or visual scene as in the SOT paradigm. Thus, condition-dependent sway is presumed to arise from internal sources of variability within the balance control system. Increased variability is presumed to indicate diminished function. Additionally, the pattern of conditions that evoke the most variability (or falls) is considered indicative of an abnormal ability to utilize information from a particular sensory system. Patterns of conditions with increased variability arise because the sensory information is not available (sensory loss), is deficient (e.g., peripheral vestibular dysfunction), or is improperly used by central mechanisms (e.g., inability to up-weight the use of vestibular cues during an eyes-closed stance on foam test condition that requires reliance on vestibular information for balance).

The mTBI participants evaluated in our study demonstrated balance deficits, quantified with conventional SOT methods (Table 1), that were similar to military populations with persistent mTBI symptoms and similar to athletic populations acutely recovering from mTBI (13, 47, 48). Composite scores on the SOT typically return to pre-injury levels within 5–10 days of the injury for collegiate athletes (10, 49). This suggests that our population had ongoing balance impairments from their most recent mTBI which occurred more than 3 months ago and, for some, several years ago. Our results that quantified condition-dependent sway using remnant sway measures were in qualitative agreement with SOT results in that the average SOT composite score was lower (worse performance) in mTBI than HC subjects and our remnant sway and internal sensory noise measures were greater (worse performance) in all 4 of our test conditions.

Remnant sway measures from the CSMI analysis are most closely related to condition-dependent sway measures commonly used in other studies since they both are presumed to measure sway variability that arises from variability (i.e., imprecision or noise) that is internal to the balance system and they both are expected to be influenced by the dynamic characteristics of the balance system [Supplemental materials; (44)]. To obtain an internal variability measure that was not influenced by balance control system dynamics, we derived our internal sensory noise measure using the results from van der Kooij and Peterka (44) that concluded that internal sensory noise was the dominant contributor to the remnant sway measure. Thus, the internal sensory noise measure provided a measure of imprecision of the balance control system that removed the influence of dynamics of the balance system. The greater internal sensory noise values seen, on average, in mTBI compared to HC subjects (Figure 2C, Table 2) indicated that head injury introduced an added imprecision into the processing of information needed for balance control.

The variation in internal sensory noise measures across the different stimulus conditions (Figure 2C, Table 2) provides some insight into how the availability of accurate sensory orientation information affects internal noise. Specifically for both groups, the internal noise was largest in the SS/EC condition when only proprioceptive and vestibular sensory systems were contributing to balance control, internal noise was lower when two sensory sources were providing accurate (SS/EO and VS/EO) or congruent (SS+VS/EO) orientation information, and the internal noise was lowest in the VS/EO condition where both proprioception and vestibular information were accurate and the visual contribution to balance control was low (low visual weight, Table 3).

### Determinants of stimulus-evoked sway

Stimulus-evoked sway provides a measure of the sensitivity to an externally applied balance perturbation with a greater sensitivity being potentially indicative of abnormal function. Increased sensitivity to a stimulus relative to control subjects is often equated with greater reliance on orientation cues provided by the sensory modality being stimulated and, thus, has been equated to measures of sensory weighting. Specifically, stimulus-evoked sway measures have been interpreted to indicate visual-vestibular impairment in mTBI subjects because they showed increased stimulus-evoked sway for both visual and galvanic vestibular stimulation (19).

However, our CSMI analysis revealed that, in addition to sensory weighting, increased sensitivity was also facilitated by diminished motor activation stiffness (e.g., Figure 4). Thus, CSMI analysis provided the ability to separately evaluate the contributions to stimulus-evoked sway from sensory integration effects, determined by model-estimated sensory weights, and from motor activation effects, determined mainly by the stiffness factor. The relative contributions of sensory integration and motor activation to response sensitivity depended on the test condition (Figure 5 and Table 4) with sensory integration weights having the largest effect on response sensitivity in the VS/EO condition and motor activation stiffness having a larger influence than sensory weights in the other three conditions. The ability to separate these influences on stimulus-evoked sway allowed for a better understanding of the mechanisms contributing to the differences between mTBI and HC subjects.

### No evidence for vestibular sensory integration abnormalities in people with chronic mTBI

The two CSMI test conditions (SS/EC and SS+VS/EO) that provided estimates of the vestibular contribution to balance control, showed that the mean values of vestibular weights were about 3% lower in mTBI than HC subjects but this difference was not significant (Table 3). However, if the vestibular contribution to balance had been judged based on the magnitude of the stimulus-evoked sway in these two conditions then mTBI subjects would have been considered to be less reliant on vestibular cues for balance than HC subjects since mTBI subject stimulus-evoked sway was significantly larger in both conditions. That is, qualitatively this increased stimulus-evoked sway could have been interpreted as indicating an over-reliance on proprioception, in the SS/EC condition, and over-reliance on combined proprioception + vision, in the SS+VS/EO condition, and therefore, a decreased reliance on vestibular information in both conditions that was possibly due to some form of vestibular dysfunction. However, the quantitative results from the CSMI analysis indicated that this qualitative interpretation of a decreased vestibular contribution was not correct. Rather, the increased sensitivity to the applied stimuli was attributable to decreased motor activation as represented by mTBI subjects having reduced stiffness and damping (Table 3). Essentially, the reduced motor activation allowed the destabilizing torque due to gravity to exert a greater influence on the magnitude of stimulus-evoked sway.

Reduced motor activation could account for previous results that showed greater sensitivity to vestibular evoked sway in response to galvanic vestibular stimulation in mTBI compared to HC subjects (19). Additionally, that study did not see the expected intra- and inter-modal sensory re-weighting behavior where an increased stimulus amplitude in one sensory modality was expected to reduce reliance (weighting) on that modality while increasing reliance on a different sensory modality (50). The inability to detect this re-weighting behavior may have been because the magnitude of stimulus-evoked sway was dominated by motor activation properties which masked changes in evoked sway caused by sensory re-weighting.

### Increased reliance on vision

The chronic mTBI group had significantly higher visual weights relative to healthy controls during the VS/EO CSMI condition (Table 3) and demonstrated significantly larger stimulus-evoked CoM sway, remnant sway, and internal sensory noise compared to the HC group. As discussed in the previous section, we consider the visual weight measures obtained from CSMI analysis to provide a more accurate assessment of the visual contribution to balance than the magnitude of visual-evoked CoM sway. However, in this case, both measures indicated significantly increased reliance on the visual stimulus. Unlike the assessment of the vestibular contribution using responses in the SS/EC and SS+VS/EO conditions, the motor activation parameters in the VS/EO condition were similar for mTBI and HC subjects and, therefore, did not play a major role in inflating the magnitude of visual-evoked CoM sway.

Other studies using different posturography assessments have shown people at various recovery times following mTBI with increased postural sway relative to healthy controls when exposed to visual scenes with motion or visual surrounds that moved (13, 15, 16, 19). Balance control requires multisensory processing and it has been theorized that visual-vestibular sensory processing may be impaired acutely after mTBI (51, 52). Disruption of multisensory processing immediately following mTBI may promote an increased reliance on visual cues that persists in people with mTBI dizziness symptoms that have not resolved (52, 53).

Recent functional brain imagining work has demonstrated evidence for this theory; people with post-mTBI visual motion sensitivity had increased brain activation in visual-vestibular multisensory processing areas when exposed to visual scenes with motion while in the MRI (52). This suggests that people with post-mTBI visual motion sensitivity have increased reliance on visual information in the visual-vestibular network subacutely after injury, and our study helps to corroborate this notion in the chronic mTBI subjects assessed through a novel standing balance test. However, our results indicate that disruption of the visual-vestibular network was not accompanied by a decreased reliance on vestibular cues.

Application of CSMI testing early post-mTBI and then across time could be used to determine whether there is evidence for early reduced utilization of vestibular information for balance that later recovers vs. other explanations that involve changes in time delay and motor activation.

### Increased time delay as an important mTBI abnormality

Increased time delay as a consequence of brain injury may be a particularly important factor affecting balance control in a subset of mTBI subjects with chronic imbalance complaints. Previous studies have shown that the mTBI population has longer response times on computerized neurocognitive evaluations (54), on a clinical assessment of catching a falling ruler (55, 56), during a simulated driving task (57), and in more functional movement tasks such as stabilizing balance after landing on a single limb (58), and performing a change of direction after a jump landing (59).

Potentially consistent with these previous studies, the time delay parameter identified using the CSMI analysis was, on average, larger in mTBI compared to HC subjects across all of the test conditions (Table 3). Additionally, there was a subset of 30% to 49% of mTBI subjects whose time delays were greater than the 90^th^ percentile time delay value of HC subjects (Table 5). Furthermore, a combination of low normalized stiffness measures and long time delays further identifies a subgroup of 22% to 39% of mTBI subjects that were largely separable from HC subjects. The existence of this subgroup and the significant correlation between normalized stiffness and time delay in all test conditions (Figure 7) led us to postulate a causal reason for this correlation that has important consequences for balance control.

**Figure 9:**
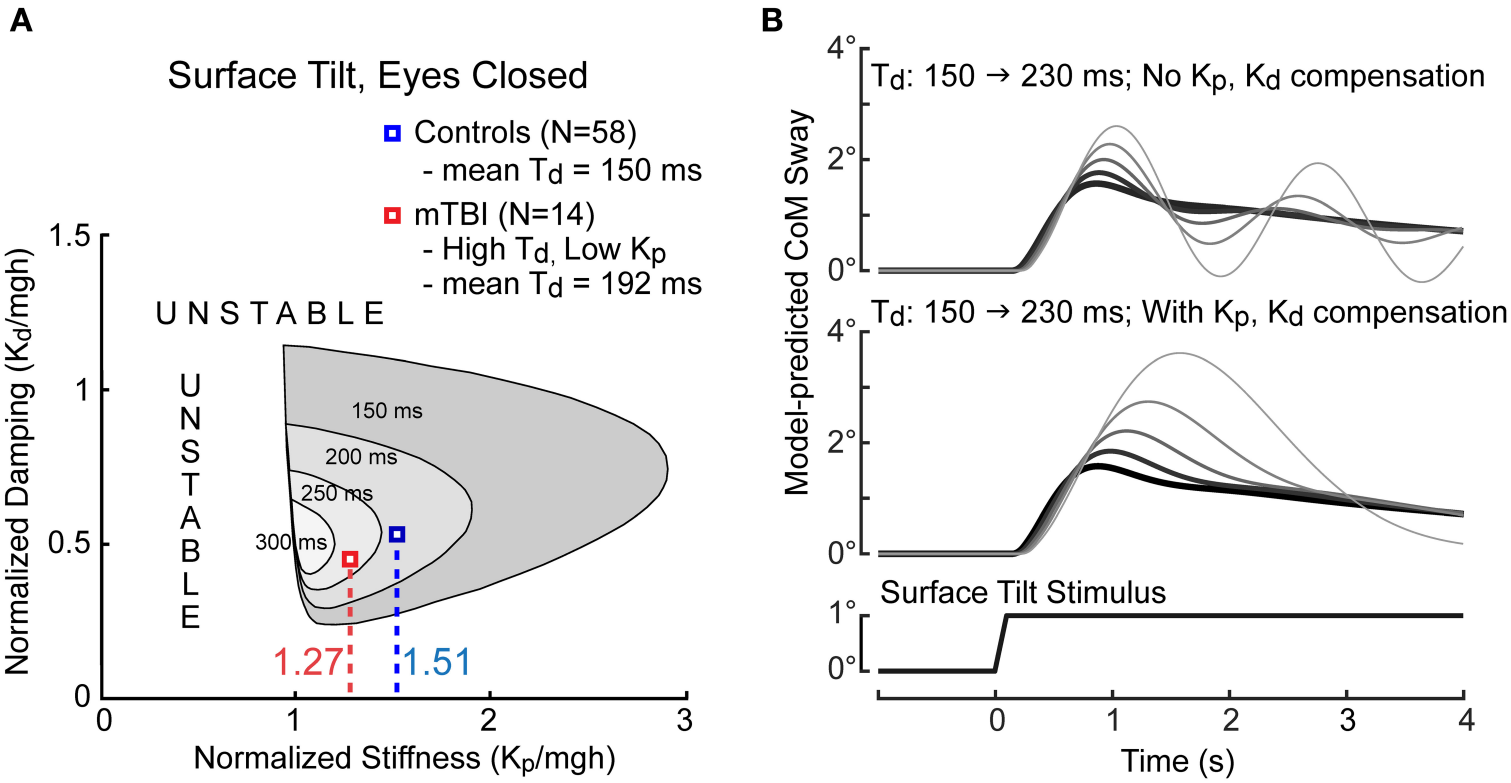
Illustration of constraints on balance system stability as a function of time delay and motor activation normalized stiffness and damping parameters. **(A)** The CSMI model-predicted regions of stability (gray regions) shrink with increasing system time delay. The mean normalized stiffness values for control subjects (blue) and for the 14 mTBI subjects (red) with particularly long time delays and low stiffness in the SS/EC condition are labeled. **(B)** Model simulations of sway responses to 1° tilts of the stance surface show the effects on the sway response as time delay increased from 150 ms (thickest dark trace) to 230 ms in increments of 20 ms in conditions where stiffness and damping parameters remained fixed (upper graph) and when normalized stiffness and damping were decreased to compensate for the increasing time delay (lower graph). Prominent oscillatory sway behavior is eliminated by lowering stiffness and damping when time delays are larger but at the expense of having a larger peak sway responses. Simulations performed using MATLAB Simulink version R2019b.

We postulate that the reduced motor activation represents a compensatory strategy implemented by the balance control system of mTBI subjects to compensate for increased time delay. Longer time delays in a feedback control system are detrimental to stability of the system. This can be seen in the illustration in Figure 9A that shows that for the CSMI model (Figure 1) the ranges of motor activation stiffness and damping parameters that are compatible with stability shrinks as the time delay of the balance system increases. One method to compensate for an increased time delay is to reduce motor activation stiffness and damping factors. The mean normalized stiffness and damping parameters are shown in Figure 9A for HC subjects and for the low stiffness – long time delay mTBI subgroup for the SS/EC condition. The location of the HC stiffness and damping parameters are well within the stability boundary for a mean HC time delay of 150 ms. The lower mean stiffness and damping values for the mTBI subgroup also ensure stability by staying well within a shrunken stability boundary defined by their 192 ms time delay (their stability boundary would be slightly larger than the region shown for a 200 ms delay).

The consequence of reduced motor activation is increased sensitivity to balance perturbations. To appreciate the consequences of compensating vs. not compensating for increased time delay, Figure 9B show CSMI model simulations of CoM sway responses to a 1° surface tilt as the time delay is increased without stiffness and damping changes (top) or with compensation (bottom) with compensatory stiffness decreases dictated by the regression relation shown in Figure 7 (and similar proportional decreases in the damping parameter). Without compensation, the balance system response became oscillatory with increasing time delay and was close to instability at the 230 ms delay. With compensation, oscillatory dynamics were avoided but at the cost of increasing peak sway amplitudes.

The reduced motor activation, while ensuring stability of the balance system, is detrimental for another reason. Specifically, reduced motor activation also enhances the sway variability due to internal sensory noise (Figure 6). Compared to HC subjects, mTBI subjects had larger magnitudes of internal sensory noise (Table 2). Additionally, about half of the low stiffness – long time delay mTBI subgroup also had high levels of internal sensory noise (Table 5). These results suggest that this subset of the low stiffness – long time delay subgroup of about 14% of mTBI subjects would be particularly vulnerable to loss of balance since peak body sway following an external perturbation depends on both the magnitude of the perturbation and the additive influence of sway variability due to internal sensory noise.

The application of experimental methods that are the same as or similar to the CSMI methods we used have revealed other subject and patient groups showing a pattern of reduced motor activation (mainly reduced stiffness) and increased time delay. Wiesmeier et al. (27) showed this pattern in healthy elderly compared to younger subjects. Pasma et al. (21) showed this pattern in healthy elderly compared to younger subjects and also in cataract patients compared to healthy elderly, in polyneuropathy patients compared to healthy elderly, and in elderly with reported impaired balance compared to healthy elderly. Overall these results imply that a number of the chronic mTBI subjects we evaluated had balance control limitations that were quite similar to elderly subjects and subjects with additional age-related deficits such as cataracts and polyneuropathy. However, elderly subjects also showed reduced vestibular contributions to balance control that were not seen in our mTBI subjects. In contrast, this low stiffness – long time delay pattern was not seen in subjects with bilateral vestibular loss (6, 51), in subjects with unilateral vestibular loss compared to age-matched controls (23), in patients with acute vestibular dysfunction (25), and in patients with Parkinson's disease compared to age-matched controls (24).

### CSMI results provide partial explanation for aphysiologic SOT pattern

Literature on the SOT indicated that certain patterns of equilibrium scores across the 6 SOT conditions appear inconsistent with expected patterns based on sensory system deficits (e.g., vestibular deficit pattern) or on deficits that affected utilization of sensory cues (e.g., visual preference pattern) (41). The unknown physiological origin led to establishment of the Aphysiological classification of SOT results with this pattern being generally characterized by poorer than expected performance on easier test conditions (Conditions 1 and 2) and, given the poorer performance on the easier conditions, better than expected performance on harder test conditions (Conditions 5 and 6). The Aphysiologic pattern may imply that a subject is a potential malingerer. However, the study that defined the Cevette criteria that we applied to identify subjects with the Aphysiologic pattern indicated that multiple disorders other than true malingering were associated with the Aphysiolgic pattern including somatoform, anxiety, and depressive disorders (42). Additionally, a later study advocated for application of a wider set of criteria to identify subjects who were Aphysiologic (meaning they were likely true malingerers) (60). When that study applied their wider set of criteria only about 10% of subjects classified as Aphysiologic using the Cevette criteria remained Aphysiologic. More recent evidence indicated that patients with “persistent postural-perceptual dizziness” (PPPD) showed a pattern on SOT equilibrium scores that was similar to that seen in our mTBI subjects as well as in patients with chronic subjective dizziness (CSD) (61, 62). Thus, we consider that the Aphysiologic pattern identified in our mTBI subjects (Supplementary Table S1) did not imply that these subjects were exaggerating balance performance but rather represented an opportunity to determine if CSMI results could give insight into causes of their poor balance control that cannot be explained by traditional expectations based on sensory deficits and sensory preferences.

Application of the Cevette criteria (42) showed that 45% on the mTBI subjects had an Aphysiologic pattern (Supplementary Table S1) with generally poorer performance across all SOT conditions compared to the mTBI group as a whole and to HC subjects (Figure 8). Of interest was whether results from CSMI testing could identify factors affecting balance control that may provide a physiological explanation for balance performance identified as Aphysiological on SOT. On the 3 CSMI tests that included surface tilt stimuli, the CSMI motor activation parameters had significantly lower values of motor activation (stiffness and damping) and longer time delay values in Aphysiologic mTBIs than in HCs (Supplementary Table S3). On the VS/EO CSMI test the visual sensory weight was larger and the time delay was longer in Aphysiologic mTBI subjects compared to HCs. Finally, on all CSMI tests, the identified internal sensory noise measure was larger in the Aphysiologic mTBI subjects (Supplementary Table S2). The generally lower motor activation and higher internal noise are factors that predict greater overall body sway in conditions when no stimulus is present and are thus consistent with dysfunctional performance where sway is elevated across all SOT conditions. As discussed previously increased time delay may be an important factor that drives a compensatory lowering of motor activation such that 22% to 39% of mTBI subjects were in a low stiffness – long time delay subgroup that distinguished them from HCs and other mTBI subjects (Table 5). A substantial fraction of this subgroup ranging from 50% to 71% were classified as Aphysiologic (Supplementary Table S1) and showed a pattern of SOT results similar to mTBI subjects classified as Aphysiologic (Figure 8). Overall, the application of CSMI methods identified physiological factors of the balance control system that can at least partially account for the Aphysiological pattern observed on the SOT in chronic mTBI subjects, suggesting that the aphysiological term may not be appropriate in some situations. Future applications of CSMI methods may similarly provide insight into unusual patterns of balance control seen in patients with disorders categorized as “functional dizziness” such as PPPD, CSD (63).

### Limitations

The CSMI model we used simplified the complexities of the actual human balance control system since the model assumed the body is a single segment inverted pendulum rather than a multi-segment system requiring multi-joint control. Other simplifications include an absence of components representing joint torques from passive properties of muscle/tendon complexes and due to rapid-acting stretch reflexes. Both of these components could contribute to stiffness-related and damping-related joint torques in the overall system. To the extent that they did contribute to overall joint torques, not including them in the model would likely bias the estimated time delay toward shorter values since both of these excluded components act more rapidly than the longer delays associated with central sensory integration and processing for motor command generation. Additionally, the identified stiffness and damping factors would be expected to include the effects of both longer and shorter-acting sources of corrective torques. A previous study that constrained the body to sway as an inverted pendulum, identified FRFs over wider bandwidth than the current study, and did include model components representing passive muscle tendon properties found that they contributed about 15% of the overall ankle torque (6). But for practical purposes, our tests were performed freestanding using shorter duration stimuli with narrower stimulus bandwidths. Attempts to use a more complex model with additional parameters led to unreliable estimates of most of the model parameters.

The physiological mechanisms needed for standing balance control differ from mechanisms required for balance during dynamic tasks such as walking (64) with unknown overlap between these tasks. Therefore, it is unclear if abnormalities in standing balance control that we identified in mTBI subjects would also impede their balance function in dynamic tasks that are arguably more relevant for daily life function (65).

Inclusion and exclusion criteria may have introduced selection bias into our study population. Inclusion in the study depended on self-reported balance issues which may have led to a lower than average SOT score. Furthermore, we excluded people with more severe cognitive impairment which may have biased our study sample. There is a known relationship between balance and cognition (66, 67). The chronicity of non-resolving mTBI symptoms experienced by participants within our study may explain the low SOT composite scores when compared to studies using more acutely (<3 months) injured people who likely recover more quickly after injury (13, 49). Patients with chronic non-resolving symptoms may have received rehabilitation. However, previous rehabilitation history was not obtained for our study. Not all underlying patient factors were captured that may explain the pronounced balance impairments observed in our cohort. For example, our participants were not screened for a history of pre-existing migraine or vestibular migraine, which has been shown to associate with balance deficits (68–70).

## Conclusion

Our CSMI methods considered the sensory, time delay, and motor aspects of the balance control system and suggest chronic mTBI-related balance deficits primarily arise from two general problems – longer time delays and reduced stiffness – and one condition-dependent problem – greater reliance on vision when viewing a perturbing visual stimulus. Contrary to popular nomenclature that attributes imbalance to vestibular origins, these results support recent evidence that balance impairments in chronic mTBI are not the result of damaged peripheral vestibular organs (7). Rather, balance deficits involve critical interactions of central processes that coordinate sensory integration and the generation of appropriate motor responses. These complex interactions require central processing time that is extended in a subset of mTBI subjects. Overall, these results suggest that rehabilitation programs focused solely on sensory aspects of balance control may be incomplete; the longer time delays and lower stiffness in participants with mTBI support an added emphasis on rapid and vigorous responses to balance perturbations.

## Data availability statement

The raw data supporting the conclusions of this article will be made available by the authors, without undue reservation.

## Ethics statement

The studies involving human participants were reviewed and approved by Oregon Health and Science University and Veterans Affairs Portland Health Care System joint Institutional Review Board. The patients/participants provided their written informed consent to participate in this study.

## Author contributions

LK and RP contributed to the conception and design of this study. KC, LP, PF, and RP contributed to data acquisition. KC, LK, LP, PF, and RP contributed to analysis and interpretation of data. KC, PA, and RP wrote the first draft of the manuscript. All authors contributed to the manuscript revision and read and approved the submitted version.

## Funding

This work was supported by the Assistant Secretary of Defense for Health Affairs (Award Nos. W81XWH-15-1-0620 and W81XWH-17-1-0424). Opinions, interpretations, conclusions, and recommendations are those of the author and are not necessarily endorsed by the Department of Defense.

## Conflict of interest

The authors declare that the research was conducted in the absence of any commercial or financial relationships that could be construed as a potential conflict of interest.

## Publisher's note

All claims expressed in this article are solely those of the authors and do not necessarily represent those of their affiliated organizations, or those of the publisher, the editors and the reviewers. Any product that may be evaluated in this article, or claim that may be made by its manufacturer, is not guaranteed or endorsed by the publisher.
