# Supplementary material for: Central sensorimotor integration assessment reveals deficits in standing balance control in people with chronic mild traumatic brain injury
Source: Front Neurol. 2022 Oct 21;13:897454. doi: 10.3389/fneur.2022.897454 (PMC9634071; doi:10.3389/fneur.2022.897454)
Supplement: Supplementary file 1 [file Table_1.DOCX]

Supplementary Material

**1. Central Sensorimotor Integration (CSMI) model equations**

The block diagram of the CSMI model in **Figure 1A** of the paper can be expressed as a differential equation that determines the body sway, *BS*, relative to Earth vertical as a function of the support surface, *SS*, stimulus and/or the visual scene, *VS*, stimulus under steady-state conditions when all transient responses that occur at stimulus initiation have decayed to negligible amounts. When all the dynamic elements of the model (which include the inverted pendulum body, *B*, the ‘motor activation’ component, *MA*, ‘torque feedback’, *TF*, and ‘time delay’, *TD*) are expressed in the Laplace domain, the equations relating *BS* to *SS* (in both eyes open and closed conditions) and/or *VS* can be solved algebraically to define ‘transfer functions’, *H*, that express the dynamic relationship between the stimulus and the body sway response:

$H_{SS to BS}=\frac{W_{prop}\cdot MA\cdot TD\cdot B}{1-TF\cdot MA\cdot TD+MA\cdot TD\cdot B}$ (S1)

$H_{VS to BS}=\frac{W_{vis}\cdot MA\cdot TD\cdot B}{1-TF\cdot MA\cdot TD+MA\cdot TD\cdot B}$ (S2)

$H_{SS+VS to BS}=\frac{\left( W_{prop}+W_{vis} \right)\cdot MA\cdot TD\cdot B}{1-TF\cdot MA\cdot TD+MA\cdot TD\cdot B}$ (S3)

With:

$B=\frac{1}{J\cdot s^{2}-mgh}$ (S4)

$MA=K_{p}+K_{d}\cdot s$ (S5)

$TD=e^{-T_{d}\cdot s}$ (S6)

$TF=\frac{K_{t}}{s}$ (S7)

where ‘*s*’ is the Laplace variable. Substituting S4 – S7 into S1 – S3 and setting $s=j2\pi f$, where *j* is the imaginary number $\sqrt{-1}$, allows for the calculation of *H* values as a function of the sinusoidal stimulus frequency, *f*. All transfer function equations assume that the sum of all sensory weights contributing to balance, in a given condition, sum to 1 meaning the value of a sensory weight represents the relative contribution of a sensory system to balance control. For example, in the eyes open surface stimulus condition proprioception, visual, and vestibular cues are the contributors to balance control. The curve fitting procedure will estimate the value of *W_prop_* for this condition and then the vestibular plus visual contribution is given by *W_vest_* + *W_vis_* = 1 – *W_prop_*.

The value of *H*, at any particular frequency, *f*, is a complex number that can be expressed in terms of a ‘magnitude function’ $\left| H\left( j2\pi f \right) \right|$ equal to the square root of the sum of squared values of the real and imaginary components of *H*, and ‘phase function’ $\angle H\left( j2\pi f \right)$equal to the arc tangent of the imaginary divided by the real components of *H*. The transfer function magnitude is also referred to as the system ‘gain function’ since it represents the body sway response magnitude normalized by the magnitude of the stimulus at each frequency value.

The free parameters are adjusted to optimally account for the frequency response function, FRF, derived from the experimental body sway responses to the pseudorandom stimulus used in the experiment (1-3). The free parameters include the sensory weight (*W*), motor activation ‘stiffness’ parameter (*K_p_*) and ‘damping’ parameter (*K_d_*), time delay (*T_d_*), and torque feedback (*K_t_*). The body moment of inertia about the ankle joint, *J*, body mass, *m*, (excluding the feet), and body center-of-mass height above the ankle joints, *h*, are derived from direct measurement of body mass and based on anthropometric body measures (4), and *g* is the gravity constant. Examples of experimental FRFs and calculated FRFs derived from transfer function equations with optimally adjusted parameters are shown in **Figure 1B**.

**2. Internal sensory noise calculation**

The above methods can be used to calculate transfer functions between any signal considered to be an input and another signal considered to be the output. In particular, the **Figure 1A** CSMI model includes a ‘sensory noise’ input signal that is an additional input at the summing junction that adds together all signals from sensory systems, including the torque feedback pathway. This sensory noise input represents a theoretical source of variability that produces the ‘remnant sway’, which is the sway variability that is not accounted for by the response to the external stimulus which is estimated by calculating the mean response to the stimulus. While there are sources of sway variability other than sensory noise (e.g., motor noise) previous results indicated that sensory noise is the dominant contributor to remnant sway in the balance control system under similar conditions (5).

If it is assumed that the remnant sway is due to internal sensory noise, then it is possible to compute properties of the internal noise given measurements of the remnant sway and knowledge of the transfer function between the sensory noise input and the body sway output. For all experimental conditions (i.e., independent of the specific type of external stimuli applied), the transfer function relating sensory noise to body sway is given by:

$H_{SN to BS}=\frac{MA\cdot TD\cdot B}{1-TF\cdot MA\cdot TD+MA\cdot TD\cdot B}$ (S8)

All the parameters of this transfer function are known based on the optimal fit to the experimental FRF relating the external stimulus to the evoked body sway in a particular experimental trial. If the power spectrum of the input to a system, *P_i_(f)*, is known then the power spectrum of the system output, *P_o_(f)* is given by:

$P_{o}\left( f \right)=P_{i}\left( f \right)\cdot\left| H_{i to o} \right|^{2}$ (S9)

where *H_i to o_* is the transfer function relating the input, *i*, to the output signal, *o*. In our application *P_i_(f)* in the sensory noise power spectrum, *P_sn_(f)*, *P_o_(f)* is the remnant sway power spectrum, *P_rem_(f)*, and $H_{i to o}$ is given by equation S8. Therefore:

$P_{sn}\left( f \right)=P_{rem}\left( f \right)\cdot\frac{1}{\left| H_{SN to BS} \right|^{2}}$ (S10)

where $P_{rem}\left( f \right)$ is the frequency domain variance about the mean body sway response to the external stimulus (6):

$P_{rem}\left( k\cdot\Delta f \right)=\frac{K_{sf}}{\left( M-1 \right)}\sum_{i=1}^{M} \left| {bs}_{i}\left( k \right)-\bar{bs\left( k \right)} \right|^{2}$ (S11)

where *bs_i_(k)* is the one-sided discrete Fourier transform of the *i^th^* cycle of the body sway signal at the *k^th^* harmonic component, *M* is the number of pseudorandom stimulus cycles averaged, $\Delta f$ is the frequency spacing between harmonic components, which is the inverse of the duration of each stimulus cycle, $\bar{bs\left( k \right)}$ is the mean discrete Fourier transform across all cycles, and *K_sf_* is a factor that appropriately scales the power spectrum such that the area under the power spectrum is equal to the mean squared value of the signal. Specifically, *K_sf_* is the inverse of the product of two times the time series sampling rate times the number of samples per stimulus cycle.

To summarize and add specific details, stimulus-response parameters of the CSMI balance control model were calculated by adjusting the parameters of the balance control model to optimally account for the experimental FRF of an individual trail. These parameters were used to calculate the $H_{SN to BS}$ transfer function (equation S8) over the frequency range $f$ = 0.05 Hz (inverse of the cycle duration of 20s) to 1.5 Hz (the upper frequency of the FRF data used in the model fit). $P_{rem}\left( k\cdot\Delta f \right)$ was calculated according to equation S11 across the 0.05 to 1.5 Hz frequency range. The frequency sampled internal sensory noise power spectrum $P_{sn}\left( k\cdot\Delta f \right)$ was calculated using equation S10 over the same 0.05 to 1.5 Hz frequency range with 30 frequency components over this range. Then the RMS value of the internal sensory noise was calculated:

${RMS}_{SN}=\sqrt{\Delta f\cdot\sum_{k=1}^{30} P_{sn}\left( k \right)}$ (S12)

**3. Supplementary Tables**

**TABLE S1:** Number of healthy control (HC) and chronic mild traumatic brain injury (mTBI) subjects with Cevette et al. (7) SOT classifications of Normal (C-N), Aphysiologic (C-A), and Vestibular Dysfunction (C-V), subjects with a combination of long time delay and normalized stiffness (*T_d_K_p_*) Central Sensorimotor Integration (CSMI) test parameters, and subjects with both C-A and *T_d_K_p_* classifications (C-A&*T_d_K_p_* ) across 4 CSMI test conditions.

| **CSMI Condition** | **HC** | | | | | | **mTBI** | | | | | |
| --- | --- | --- | --- | --- | --- | --- | --- | --- | --- | --- | --- | --- |
|  | N | C-N | C-A | C-V | *T_d_K_p_* ^a^ | C-A&*T_d_K_p_* | N | C-N | C-A | C-V | *T_d_K_p_* ^a^ | C-A& *T_d_K_p_* |
| **SS/EC** | 58 | 52 | 2 | 4 | 1 | 0 | 49 | 24 | 22 | 3 | 14 | 10 |
| **SS/EO** | 58 | 52 | 2 | 4 | 2 | 1 | 50 | 24 | 23 | 3 | 11 | 6 |
| **VS/EO** | 58 | 50 | 1 | 4 | 2 | 0 | 46 | 24 | 19 | 3 | 10 | 5 |
| **SS+VS/EO** | 58 | 52 | 2 | 4 | 4 | 1 | 49 | 24 | 22 | 3 | 19 | 13 |
| a – classification based on time delay values that were greater than 90th percentile values and normalized stiffness values that were less than 10th percentile values derived from HC data.  Abbreviations: N, number of participants; EC, eyes closed; EO, eyes open; SS, support surface stimulus; VS, visual surround stimulus. | | | | | | | | | | | | |

**TABLE S2:** RMS values of stimulus-evoked CoM sway, remnant CoM sway, and internal sensory noise for healthy controls (HC) and chronic mild traumatic brain injury (mTBI) groups with SOT performance classified as Not Aphysiologic and Aphysiologic. Outcomes presented as means and standard deviations.

|  | **HC** | **mTBI** | | |
| --- | --- | --- | --- | --- |
| **CSMI Condition and Measure** | **n =58** | | **Not Aphysiologic^a^ (n = 27)** | **Aphysiologic^a^**  **(n = 25)** |
| SS/EC |  | |  |  |
| N completing condition | 58 | | 27 | 22 |
| Stimulus-evoked CoM Sway | 0.804 (0.160) | | 0.897 (0.222) | 1.067 (0.370)*^ |
| Remnant CoM Sway | 0.416 (0.124) | | 0.491 (0.138) | 0.796 (0.526)*^ |
| Internal Sensory Noise | 0.141 (0.029) | | 0.153 (0.039) | 0.220 (0.145)*^ |
| SS/EO |  | |  |  |
| N completing condition | 58 | | 27 | 23 |
| Stimulus-evoked CoM Sway | 0.494 (0.140) | | 0.576 (0.192) | 0.724 (0.300)*^ |
| Remnant CoM Sway | 0.301 (0.129) | | 0.418 (0.152) | 0.678 (0.441)*^ |
| Internal Sensory Noise | 0.107 (0.036) | | 0.135 (0.036) | 0.190 (0.130)*^ |
| VS/EO |  | |  |  |
| N completing condition | 55 | | 27 | 19 |
| Stimulus-evoked CoM Sway | 0.188 (0.088) | | 0.298 (0.134)* | 0.449 (0.253)*^ |
| Remnant CoM Sway | 0.280 (0.126) | | 0.467 (0.232)* | 0.682 (0.429)*^ |
| Internal Sensory Noise | 0.070 (0.029) | | 0.099 (0.042)* | 0.147 (0.087)*^ |
| SS+VS/EO |  | |  |  |
| N completing condition | 58 | | 27 | 22 |
| Stimulus-evoked CoM Sway | 0.892 (0.159) | | 1.001 (0.224) | 1.189 (0.329)*^ |
| Remnant CoM Sway | 0.356(0.102) | | 0.468 (0.124)* | 0.712 (0.390)*^ |
| Internal Sensory Noise | 0.118 (0.023) | | 0.140 (0.034) | 0.188 (0.099)*^ |
| All sway and internal noise measures have units of degrees.  a – Not Aphysiologic and Aphysiologic groups were determined using SOT data based on criteria defined in Cevette et al. (7).  * indicates a Tukey adjusted significant difference to HC from a General Linear Model.  ^ indicates a Tukey adjusted significant difference to Not Aphysiologic mTBI from a General Linear Model.  Abbreviations: N, number of participants; EC, eyes closed; EO, eyes open; SS, support surface stimulus; VS, visual surround stimulus; RMS, root mean square; CoM, center of mass. | | | | |

**TABLE S3:** Central Sensorimotor Integration (CSMI) test model-derived parameters for healthy controls (HC) and chronic mild traumatic brain injury (mTBI) groups with SOT performance classified as Not Aphysiologic and Aphysiologic. Outcomes presented as means and standard deviations.

|  | **HC** | **mTBI** | |  |
| --- | --- | --- | --- | --- |
| CSMI Condition and Parameter |  | **Not Aphysiologic^a^ (n = 27)** | **Aphysiologic^a^**  **(n = 25)** |  |
| SS/EC |  |  |  |  |
| N completing condition | 58 | 27 | 22 |  |
| Proprioceptive Weight | 0.509 (0.085) | 0.504 (0.068) | 0.545 (0.075) |  |
| Vestibular Weight | 0.491 (0.085) | 0.496 (0.068) | 0.455 (0.075) |  |
| Time Delay (ms) | 150 (14.0) | 159 (21) | 172 (25)*^ |  |
| Torque Feedback (rad/Nms)^b^ | -3.966 (0.186) | -4.002 (0.189) | -3.951 (0.264) |  |
| Normalized Stiffness | 1.505 (0.129) | 1.434 (0.130) | 1.389 (0.170)* |  |
| Normalized Damping | 0.531 (0.069) | 0.501 (0.046) | 0.484 (0.070)* |  |
| SS/EO |  |  |  |  |
| N completing condition | 58 | 27 | 23 |  |
| Proprioceptive Weight | 0.298 (0.051) | 0.306 (0.044) | 0.324 (0.072) |  |
| Vestibular + Visual Weight | 0.702 (0.051) | 0.694 (0.044) | 0.676 (0.072) |  |
| Time Delay (ms) | 132 (21.0) | 141 (32) | 150 (24)* |  |
| Torque Feedback (rad/Nms)^b^ | -4.015 (0.205) | -4.107 (0.265) | -4.022 (0.329) |  |
| Normalized Stiffness | 1.578 (0.231) | 1.485 (0.185) | 1.369 (0.138)* |  |
| Normalized Damping | 0.527 (0.074) | 0.490 (0.073)* | 0.445 (0.075)* |  |
| VS/EO |  |  |  |  |
| N completing condition | 55 | 27 | 19 |  |
| Visual Weight | 0.108 (0.045) | 0.139 (0.062)* | 0.169 (0.050)* |  |
| Proprioceptive + Vestibular Weight | 0.892 (0.045) | 0.861 (0.062)* | 0.831 (0.050)* |  |
| Time Delay (ms) | 200 (21.0) | 216 (27)* | 216 (28)* |  |
| Torque Feedback (rad/Nms)^b^ | -4.770 (1.496) | -4.349 (0.797) | -4.067 (0.351) |  |
| Normalized Stiffness | 1.267 (0.101) | 1.249 (0.213) | 1.184 (0.082) |  |
| Normalized Damping | 0.502 (0.056) | 0.484 (0.059) | 0.479 (0.051) |  |
| SS+VS/EO |  |  |  |  |
| N completing condition | 58 | 27 | 22 |  |
| Proprioceptive + Visual Weight | 0.552 (0.064) | 0.555 (0.075) | 0.581 (0.058) |  |
| Vestibular Weight | 0.448 (0.064) | 0.445 (0.075) | 0.419 (0.058) |  |
| Time Delay (ms) | 140.0 (18.0) | 159 (24)* | 173 (32)* |  |
| Torque Feedback (rad/Nms)^b^ | -3.986 (0.193) | -4.003 (0.183) | -3.938 (0.224) |  |
| Normalized Stiffness | 1.488 (0.134) | 1.404 (0.112)* | 1.341 (0.156)* |  |
| Normalized Damping | 0.504 (0.078) | 0.480 (0.052) | 0.447 (0.054)* |  |
| a – Not Aphysiologic and Aphysiologic groups were determined using SOT data based on criteria defined in Cevette et al. (7)  b – Mean and standard deviation of torque feedback based on log10 of parameter values.  * indicates a Tukey adjusted significant difference to HC from a General Linear Model  ^ indicates a Tukey adjusted significant difference to Not aphysiolocial mTBI from a General Linear Model  Abbreviations: N, number of participants; EC, eyes closed; EO, eyes open; SS, support surface stimulus; VS, visual surround stimulus; ms, milliseconds; rad, radians; Nms, Newton meter second | | | | |

**4. References**

1. Peterka RJ. Sensorimotor integration in human postural control. J Neurophysiol. 2002;88(3):1097-118.

2. Peterka RJ. Simplifying the complexities of maintaining balance. IEEE Engineering in Medicine and Biology Magazine. 2003;22(2):63-8.

3. Peterka RJ, Murchison CF, Parrington L, Fino PC, King LA. Implementation of a Central Sensorimotor Integration Test for Characterization of Human Balance Control During Stance. Front Neurol. 2018;9:1045.

4. Winter DA. Biomechanics and motor control of human movement: John Wiley & Sons; 2009.

5. van der Kooij H, Peterka RJ. Non-linear stimulus-response behavior of the human stance control system is predicted by optimization of a system with sensory and motor noise. J Comput Neurosci. 2011;30(3):759-78.

6. Pintelon R, Schoukens J. System identification: a frequency domain approach: John Wiley & Sons; 2012.

7. Cevette MJ, Puetz B, Marion MS, Wertz ML, Muenter MD. Aphysiologic performance on dynamic posturography. Otolaryngol Head Neck Surg. 1995;112(6):676-88.
